# Genome-Based Mining of Carpatamides I–M and Their Candidate Biosynthetic Gene Cluster

**DOI:** 10.3390/md22110521

**Published:** 2024-11-20

**Authors:** Shu-Mei Shen, Yun-Chang Xie, Li-Rong Tu, Miao-Er Wu, Yan-Min Wang, Chun-Hui Song, Yu-Hui Sun, Ming-He Luo

**Affiliations:** 1 School of Pharmacy and Bioengineering, Chongqing University of Technology, Chongqing 400054, China; shenm51627@stu.cqut.edu.cn (S.-M.S.); tlr110825@stu.cqut.edu.cn (L.-R.T.); 2 College of Life Sciences, Jiangxi Normal University, Nanchang 330022, China; xieyunchang@jxnu.edu.cn (Y.-C.X.); miaokanshijie@163.com (M.-E.W.); wym0826333@163.com (Y.-M.W.); schxzx1977@126.com (C.-H.S.); 3 School of Pharmacy, Huazhong University of Science and Technology, Wuhan 430030, China

**Keywords:** carpatamides, manumycin, natural products, genome mining

## Abstract

Chemically investigating the marine-derived *Streptomyces parvus* 1268 led to the isolation of a new compound of carpatamide I (**1**). Subsequent genomic analysis identified its candidate biosynthetic gene cluster *ctd* of approximately 44 kb. In order to obtain more carpatamide derivatives, we conducted the upregulation of Ctd14, which is a positive regulator, and obtained improvement of carpatamide I and four new compounds of carpatamides J–M (**2**–**5**). The structures of the aforementioned five new isolates were identified by a combination of ESI-HRMS as well as one-dimensional (1D) and two-dimensional (2D) spectral NMR datasets. Bioassay results showed that compounds **1**–**5** displayed anti-inflammatory activity and weak cytotoxicity against cell lines of A549, HT-29, and HepG2.

## 1. Introduction

Manumycins exhibit a broad range of biological activities [1], including cytotoxic activity [2,3], anti-inflammatory [4], and so on. Recently, manumycin polyketides were also found to be molecular glues between UBR7 and P53 [5], making them potential compounds for the development of drug leads, which have triggered great interest in compounds of this class. Carpatamides are the new members of manumycins, which were isolated from *Streptomyces maritimus* through cytotoxic activity screening, and carpatamide A and carpatamide C showed cytotoxicity against HCC366, A549, and HCC44, with IC_50_ values of 2.2–8.4 μM [6].

**Figure 1:**
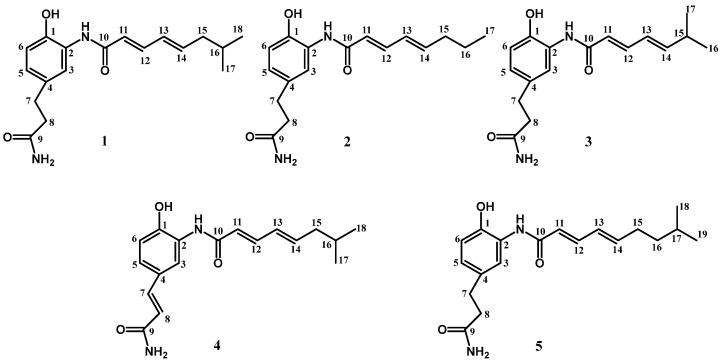
Chemical structures of the new isolates carpatamides I–M (**1**–**5**).

**Figure 2:**
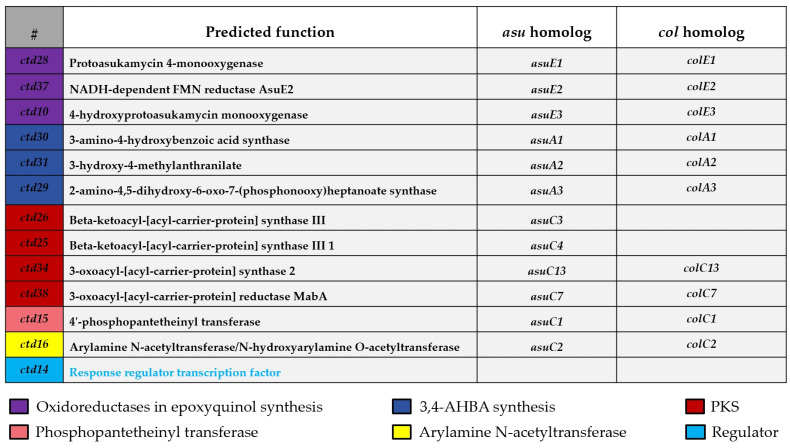
The core *ctd* genes annotations with *asu* and *col* homologs (also part of core genes of manumycin-group metabolites), along with the annotation of regulator gene of *ctd14* in *ctd*.

During our mining process for new compounds, we found that *Streptomyces parvus* 1268 could produce a compound with UV/Vis spectrum (λmax at 272 and 313 nm) in AM3 medium, which is similar to that of carpatamides [6,7]. Further chemically investigation led to the isolation of a new compound named carpatamide I (**1**) (Figure 1). Subsequent genome sequencing and analysis of *Streptomyces parvus* 1268, we found a candidate biosynthetic gene cluster (BGC) named *ctd*, showing 31% and 27% similarity to that of colabomycin E (NCBI GenBank: KF850685.1) [8] and asukamycin (NCBI GenBank: GQ926890.1) [9], respectively (Figure S1), which have the unique 3,4-AHBA synthase encoding gene of manumycin biosynthesis (Figure 2).

**Figure 3:**
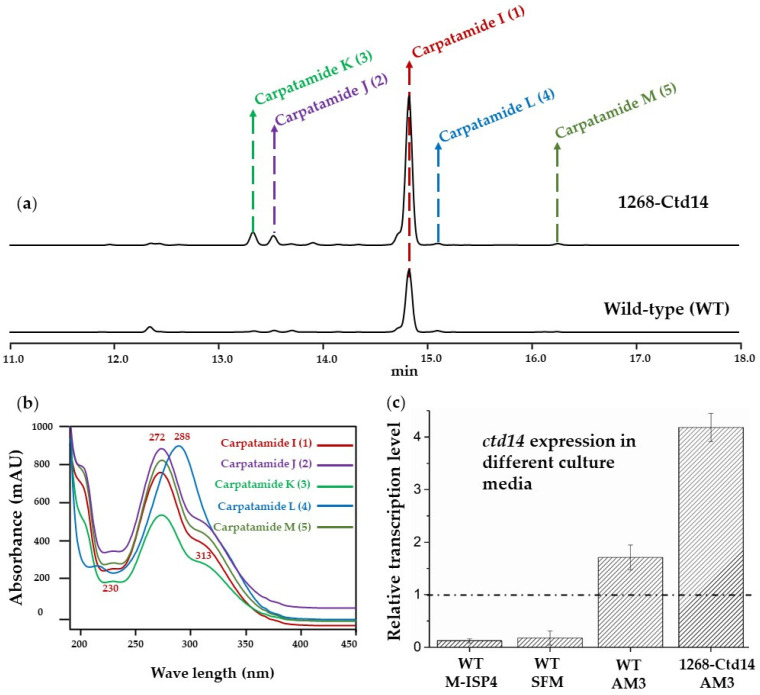
HPLC-DAD analysis results of the fermentation extracts of overexpression transformant of *Streptomyces parvus* 1268-Ctd14 (1268-Ctd14) and wild-type (WT) at 264 nm in AM3 medium (**a**). The UV/Vis spectra of compounds **1**–**5** (carpatamides I–M), while λmax at 272 and 313 nm are the UV/Vis character of compounds **1**, **2**, **3**, **5**, which is similar to that of carpatamides A and B (**b**). Gene expression analysis of *ctd14* in different culture media for WT and 1268-Ctd14 (**c**).

Meanwhile, bioinformatic analysis of *ctd* also revealed several genes encoding a series of regulators. Among them, through synchronous expression, clear positive regulatory genes can be directly selected to improve the biosynthesis of carpatamides. Therefore, by overexpressing the aforementioned positive regulatory genes in *Streptomyces parvus* 1268, a well-established and widely employed strategy for upregulating gene expression in genome mining, we effectively enhanced the further exploration of carpatamides [10,11]. The regulator gene *ctd14*, encoding the response regulator transcription factor in *ctd* (Figure 2 and Table S1), can be expressed more efficiently in the media suitable for *Streptomyces parvus* 1268 producing carpatamides rather than the counterpart hampering strain’s biosynthesis of carpatamides (Figure 3c). This contrast obviously verified that Ctd14 could be a positive regulator to support the carpatamides biosynthesis in *Streptomyces parvus* 1268. Therefore, the corresponding *ctd14* overexpressed transformant strain 1268-Ctd-14 was constructed and displayed significant improvement by not only increasing the yield of **1** but also stimulating the transformant strain to biosynthesize four new carpatamides (**2**–**5**) (Figure 3a,b).

In this study, we report the isolation, identification, and bioactivities of new carpatamides obtained by chemical investigation and upregulation of Ctd14 and the candidate biosynthetic gene cluster of carpatamides.

## 2. Results and Discussion

### 2.1. Isolation and Identification of a New Compound of Carpatamide I

Chemical investigation of *Streptomyces parvus* 1268 through a combination of silica gel column chromatography (CC) and semipreparative high-performance liquid chromatography (HPLC) led to the isolation of compound **1** from *Streptomyces parvus* 1268 (Figure 1). The structure of compound **1** was determined based on the ESI-HRMS, one-dimensional (1D), and two-dimensional (2D) spectral NMR data of compound **1**.

**Figure 4:**
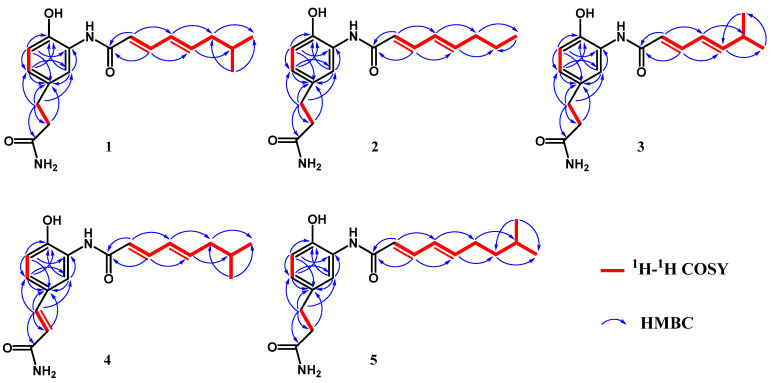
Key COSY and HMBC correlations of carpatamides I–M (**1**–**5**).

Compound **1** was obtained as a light-yellow powder with a molecular formula of C_18_H_24_N_2_O_3_ established by the ESI-HRMS with a signal of *m*/*z* 339.1682 ([M + Na]^+^) (Figure S2), indicating eight degrees of unsaturation. The ^13^C NMR and HSQC spectra unveiled the existence of two methyl, three methylene, and eight methine groups, as well as five quaternary carbons. In the ^1^H NMR spectrum, four olefinic protons at *δ*_H_ 6.22 (H-11, d, *J* = 15.2 Hz), 7.28 (H-12, dd, *J* = 15.2, 10.4 Hz), 6.28 (H-13, dd, *J* = 15.2, 10.4 Hz) and 6.16 (H-14, dt, *J* = 15.2, 7.2 Hz) indicate the presence of a conjugated *E*-configuration diene (Table 1 and Table 2). The ^1^H-^1^H COSY correlations of H-11/H-12, H-12/H-13, H-13/H-14, H-14/H-15, H-15/H-16, H-16/H-17, and H-16/H-18 further confirmed it and extent the diene to form an unsaturation aliphatic chain (“upper” chain), which is established by the HMBC correlation of H-11/C-10, C-12, C-13; H-12/C-10, C-13 C-11, C-13, C-14; H-13/C-11, C-12, C-14, C-15; H-14/C-12, C-13, C-15, C-16; H-15/C-13, C-14, C-16, C-17, C-18; H-16/C-14, C-15, C-17, C-18; H-17/C-15. C-16, C-18; H-18/C-15, C-16, C-17. In the ^1^H spectrum, the remaining three alkenyl hydrogen proton signals at *δ*_H_ 7.52 (H-3, s), 6.89 (H-5, dd, *J* = 8.4, 2.0 Hz), and 6.79 (H-6, d, *J* = 8.4 Hz), which are corresponding to the ^13^C signals at *δ*_C_ 123.5 (C-3), 126.9 (C-5), and 117.7 (C-6) established by the HSQC spectrum, respectively, unraveled a tri-substituted benzene ring moiety. This is confirmed by the HMBC correlations of H-3/C-1, C-2, C-4, C-5; H-5/C-1, C-3, C-4, C-6; H-6/C-1, C-2, C-4, C-5 and the ^13^C signals at *δ*_C_ 148.1 (C-1), 127.4 (C-2), 133.7 (C-4) (Figures S3–S7). The remaining two methylene proton signals in the ^1^H spectrum at *δ*_H_ 2.83 (H-7, t, *J* = 7.2 Hz), 2.47 (H-8, t, *J* = 7.2 Hz), and the correlations in the ^1^H-^1^H COSY and HMBC spectra of H-7/H-8; H-7/C-8, C-9; H-8/C-7, C-9, suggest the existence of a saturated “lower chain”, which is connected with the tri-substituted benzene ring unit at C-4 confirmed by the correlation of H-3/C-7, H-5/C-7; H-7/C-3, C-4, C-5; H-8/C-4 in the HMBC spectra. The carbon signal at *δ*_C_ 167.7 (C-10), along with the correlation of H-11/C-10, C-12, C-13; H-12/C-10, C-11, C-13, C14 strongly indicate that the unsaturated fatty chain of the “upper” chain is connected to the central benzene ring unit through an amide bond formed by C-10 (Figure 4 and Figures S3–S7). These characters indicate that compound **1** is a derivative of carpatamides, as shown by a comparison with the literature [6]. The exchangeable protons of 6.75, 7.28, 9.50, and 9.80 are observed when the ^1^H NMR spectrum is detected in DMSO-*_d_*_6_ (Table S2). The ^1^H-^1^H COSY correlations of 6.75 and 7.28 displayed in the DMSO-*_d_*_6_ solvent suggest that they may connected to an N atom to form an NH_2_ unit (Figures S8–S12). The chemical shift of C-1 (*δ*_C_ 146.2), C-2 (*δ*_C_ 126.2), C-5 (*δ*_C_ 124.6), C-9 (*δ*_C_ 173.5) (Table S2) in the ^13^C spectrum suggested C-1, C-2, and C-9 are substituted by -OH, -NHR, and -NH_2_, respectively. The acylated amine carbon of C-9 is confirmed by the HMBC correlations of 6.75 and 7.28 to C-9 (*δ*_C_ 173.5) detected in DMSO-*_d_*_6_ (Figures S8–S12). These are also well matched with the ESI-HRMS data of compound **1**. Thus, compound **1** is identified and named as carpatamide I.

### 2.2. Genome Sequencing and Biosynthetic Gene Cluster Analysis

Due to the absence of a gene cluster of carpatamides and our intention to utilize it to obtain more carpatamide derivatives, the genome (ID CP162609) of *Streptomyces parvus* 1268 was sequenced, which was conducted by Shanghai Biozeron Biotechnology CO., LTD, and analyzed using AntiSMASH 7.0 [12]. And we found a candidate BGC (named *ctd*) which may be responsible for the production of manumycin-group metabolites characterized by a 3,4-AHBA synthase, which is similar to that of colabomycin E [8] and asukamycin BGC [9]. A comparison of the *ctd* in the *Streptomyces parvus* 1268 with BGCs of colabomycin E and asukamycin helped to establish *ctd* boundaries (*ctd1–41*). The *ctd* shares some core genes of manumycin-group metabolites. For example, the three genes of *ctd29–31* are suggested to build 3-amino-4-hydroxybenzoic acid (3,4-AHBA) unit [8,9,13], two beta-ketoacyl-ACP synthase III genes [14] of *ctd25* and *ctd26* may be involved in the production “upper” chain of polyene PKS moiety. The KS gene of *ctd34* (3-oxoacyl-[acyl-carrier-protein] synthase 2) may be responsible for the formation of the “lower” chain. The amino-hydroxy phenyl moiety may be ligated to the “upper” side chain by the arylamine N-acyltransferase (Ctd16) [9]. Three oxidoreductase genes [15] (*ctd10*, *ctd28*, and *ctd37*) are supposed to be responsible for the formation of 5,6-epoxy-4-hydroxycyclohex-2-en-1-one moiety (Table S1 and Figure S1). However, *ctd* also has some distinct characters include genes of *ctd12* and *ctd27*, which may be involved in the formation of the NH_2_ in the “lower” chain, the absence of thioesterase gene, the existence of *ctd36*, which may function as a thioesterase, the enoyl-reductase (ER) gene (*ctd32*) which may be responsible for the saturated “lower” side chain, the lack of chain-length factor, which may explain the structure variation between carpatamides and other manumycin-type compounds.

### 2.3. Overexpression of ctd14 In Vivo to Enhance the Diversity of Carpatamides

Meanwhile, a detailed analysis of *ctd* revealed that it may encode a response regulator Ctd14, which may regulate the *ctd* expression and correspond to carpatamides’ biosynthesis by response to the surrounding signal [10,11]. To confirm the function of Ctd14, we adopted the *ctd14* expression comparison among the *Streptomyces parvus* 1268 cultivated in different media, including AM3 (with stable production of **1**) and M-ISP4/SFM (without **1** produce). The following quantitative analysis showed that the *ctd14* expression level was obviously higher in strains cultivated in AM3 than in the culture of M-ISP4/SFM (Figure 3c). This difference in gene expression indicated that Ctd14 might positively interact with the *ctd* expression to produce carpatamides. Given the effectiveness of positive regulators, overexpression of their corresponding encoded genes embedded in BGC is a practical strategy in bioactive secondary metabolites development [10,11]. Thus, in order to improve the strain’s biosynthesis of carpatamides, we further constructed the *ctd14* in situ overexpression transformant strain 1268-Ctd-14. To our delight, the subsequent fermentation of 1268-Ctd-14 not only efficiently increased the yield of **1** (Figure 3a,b) but also promoted the host to produce four new derivatives (**2**–**5**) (Figure 1 and Figure 3a,b) of carpatamides (Figure S13). Therefore, Ctd14 is identified as a novel positive regulator in carpatamide biosynthesis, enhancing not only the production of compound **1** but also diversifying the biosynthetic process to yield new carpatamide derivatives **2**–**5**.

The biosynthesis of natural products is strictly regulated in bacteria; numerous specific or pleiotropic regulators and their mechanisms have been uncovered [10,11]. Given the effectivity of positive regulators, overexpression of their corresponding encoded genes embedded in BGC is a practical strategy in bioactive secondary metabolites development. In this study, a new positive regulator, Ctd14, annotated as a response regulator, exhibits promising advancements that underscore the feasibility of utilizing a novel, valuable regulator in the exploration of carpatamide analogs.

### 2.4. Isolation of Carpatamides J–M and Their Structure Elucidation

Based on the fact that the upregulation transformant of *Streptomyces parvus* 1268-Ctd-14 could efficiently increase the yield and diversity of carpatamides, we performed the fermentation of 1268-Ctd-14 and combined its extracts. Then, these extracts were further purified by a combination of CC and HPLC as previously performed and finally obtained other four compounds of **2**–**5,** which have similar UV/Vis spectra with that of compound **1** from *Streptomyces parvus* 1268-Ctd14 (Figure 1). Their structures were determined on the basis of the ESI-HRMS, as well as 1D and 2D spectral NMR data analysis as previously.

Compound **2** has the molecular formula of C_17_H_22_N_2_O_3_ determined by its ESI-HRMS, which shows a signal at *m*/*z* 325.1521 ([M + Na]^+^) (Figure S14). The spectral NMR data is similar to that of compound **1**, with only the absence of one methyl (*δ*_H_ 0.94, *δ*_C_ 22.9) and one methine (*δ*_H_ 1.73, *δ*_C_ 29.7) signal and the addition of methylene signals of *δ*_H_ 1.49 and *δ*_C_ 23.1 (Table 1 and Table 2) in compound **2**, which indicate that the “upper” chain in compound **2** was replaced totally as a straight chain. The correlations of H-11/C-10, C-12, C-13; H-12/C-10, C-11, C-13, C-14; H-13/C-11, C-12, C-14, C-15; H-14/C-12, C-13, C-15, C-16; H-15/C-13, C-14, C-16, C-17; H-16/C-14, C-15, C-17; H-17/C-15, C-16 in the HMBC spectrum further confirmed it (Figure 4 and Figures S15–S20). Finally, compound **2** was identified and named as carpatamide J.

Compound **3** displayed a molecular formula of C_17_H_22_N_2_O_3_ on the basis of the ESI-HRMS signals at *m*/*z* 325.1534 ([M + Na]^+^) (Figure S21). A careful comparison of the spectral NMR data with that of compound **1** showed that compound **3** does not contain the methylene signal of *δ*_H_ 2.10 and *δ*_C_ 43.5 of compound **1** (Table 1 and Table 2). At the same time, the chemical shift of the methyl and methine signals in compound **3** has increased to *δ*_H_ 1.07 (H-16,17, d, *J* = 6.8 Hz), *δ*_C_ 22.3 (C-16,17) and *δ*_H_ 2.45 (H-15, m), *δ*_C_ 32.8 (C-15), respectively, which indicate that the methylene is absence and the methine is attached directly onto the diene of the “upper” chain in compound **3**. This is also confirmed by the correlations of H-14/C-15, C-16, C-17; H-15/C-13, C-14, C-16, C-17; H-16/C-14, C-15, C-17; H-17/C-14, C-15, C-16 in the HMBC spectrum of compound **3** (Figure 4 and Figures S22–S26). Therefore, the compound **3** was assigned and named carpatamide K.

Compound **4** showed the molecular formula of C_18_H_22_N_2_O_3_ determined by the ESI-HRMS signals at *m*/*z* 337.1527 ([M + Na]^+^) (Figure S27), which is two mass unit deficiency relative to compound **1**, suggesting two H atoms loss in compound **4** compared with compound **1**. Comparisons of the 1D spectral NMR data with those of compound **1** unveiled that the two methylene signals at *δ*_H_ 2.83, δ_C_ 32.3 and *δ*_H_ 2.47, *δ*_C_ 38.7 of compound **1** are absent in compound **4**, at the same time the alkene signals of *δ*_H_ 7.47, *δ*_C_ 143.0 and *δ*_H_ 6.46, *δ*_C_ 118.6 are appeared in compound **4**, suggesting that compound **4** has two H atoms deficiency between C-7 and C-8 to form a double bond (Table 1 and Table 2), which is further confirmed by HMBC correlation of H-7/C-3, C-4, C-5, C-8, C-9; H-8/C-4, C-7, C-9 in compound **4** (Figure 4 and Figures S28–S34). The *E* configuration of H-7 and H-8 is confirmed by the *J* value (15.6 Hz) between themself. Based on these, compound **4** was identified and named as carpatamide L.

The molecular formula of compound **5** was determined to be C_19_H_26_N_2_O_3_ based on the ESI-HRMS signals at *m*/*z* 353.1843 ([M + Na]^+^) (Figure S35). The ^1^H and ^13^C spectral NMR data resemble those of compound **1**, except for one additional methylene signal (*δ*_H_ 1.35, *δ*_C_ 39.2, C-16) observed in the spectrum of compound **5** (Table 1 and Table 2). The correlations of H-15/C-13, C-14, C-16, C-17, C-18; H-16/C-14, C-15, C-17, C-18, C-19; H- 17/C-15, C-16, C-18, C-19 in the HMBC spectrum of compound **5** indicates an additional methylene extension of the “upper” chain in compound **5** relative to compound **1** (Figure 4 and Figures S36–S40). Therefore, compound **5** was finally determined and named carpatamide M.

### 2.5. Bioassay of Compounds ***1***–***5***

Due to the excellent bioactivities of carpatamide A and carpatamide C, isolates in this study were evaluated for their anti-inflammatory and cytotoxic activities. Results showed that compounds **1**–**5** exhibited weak anti-inflammatory activities and cytotoxic activity against cell lines of A549, HepG2, and HT-29 with IC_50_ of 25–47 μM (Table 3). None of them showed bioactivities as interesting as carpatamides A and C. This is probably caused by the existence of the NH_2_ unit in the “lower” chain.

## 3. Materials and Methods

### 3.1. General Experimental Procedures

UV spectra were performed on a U-2600 spectrometer (Shimadzu, Tokyo, Japan). NMR spectra of compounds **1**–**4** was recorded with a Bruker Avance III HD 400 MHz (Bruker, Bremen, Germany), and NMR spectra of compound **5** was performed on Bruker Avance III HD 500 MHz (Bruker, Bremen, Germany). Chemical shifts (*δ*) are given in ppm with TMS as the reference. ESI-HRMS spectra were acquired with a Maxis quadrupole-time-of-flight mass spectrometer (Bruker, Bremen, Germany). Column chromatography (CC) was carried out with silica gel (200–300 mesh (Yantai Jiangyou Silica Gel Development Co., Ltd., Qingdao, China). Semi-preparative High-Performance Liquid Chromatography (HPLC) was implemented with a Thermo Scientific Ulti Mate 3000 (ThermoFisher, Waltham, MA, USA) with a C18 column (250 × 10 mm, 5 μm, YMC Co., Ltd., Kyoto, Japan). Natural sea salt is obtained from Guangdong Province Salt Industry Group Co., Ltd., China (Guangdong Province Salt Industry Group Co., Ltd., Guangzhou, China).

### 3.2. BGC Bioinformatic Analysis

The genome (ID CP162609) of *Streptomyces parvus* 1268 was analyzed using antiSMASH 7.0 [12] with detection strictness of “relaxed”. BGC is similar to that of colabomycin E and asukamycin and was further searched using 2ndfind (https://biosyn.nih.go.jp/2ndfind/, accessed on 18 July 2024).

### 3.3. Bacterial Strains, Plasmids and DNA Manipulation

Bacterial strains and plasmids of this study are listed in Table S3 of Supporting Information. DNA manipulations were carried out using standard procedures for *E. coli* and *Streptomyces*. All chemical reagents were obtained from Sigma-Aldrich (Sigma-Aldrich, Shanghai, China). Primers were synthesized by Tsingke (Table S4). DNA sequencing of PCR products was performed by Tsingke.

Marine-derived actinobacteria 1268 was a gift from Prof. Jianhua Ju, with the accession number PP907725. The 16S rRNA sequence analyses revealed that the strain of 1268 is a member of *Streptomyces* sp. with the closest identity (97%) to *Streptomyces parvus* NRRL B-1455 and *Streptomyces parvus* JCM 4069 (Figure S41). Thus, it was identified as *Streptomyces parvus* 1268. A voucher specimen was deposited in 20% glycerol at Chongqing University of Technology in Chongqing, China.

### 3.4. Culture and Fermentation Conditions

*Streptomyces parvus* 1268 and its transformant maintained on ABB13 plates with 3% sea salt (0.5% soytone, 0.5% soluble starch, 0.3% CaCO_3_, 0.2% MOPS, 2% agar, 3% sea salt) was firstly cultured in TSBY medium (10.5% sucrose, 3% tryptone soy broth, 0.5% yeast extract) for 2 days as seed cultures at the condition of 28 °C and 200 rpm. Then, 12 mL seed culture was transferred into a 2000 mL Erlenmeyer flask containing 600 mL AM3 medium (1.5% soluble starch, 1.5% glycerol, 1.5% bacteriological peptone, 0.5% soybean meal, 0.5% CaCO_3_, 3% sea salt). These flasks were further cultured for 7 days under the same conditions of 28 °C and 200 rpm. Ten-liter cultures were harvested in this way.

### 3.5. Overexpression of ctd14 In Vivo

The *ctd14* was amplified by primers Ctd14-up/re; then, the corresponding DNA product was purified and digested with *Nde*I/*Xba*I. The enzymatically digested DNA fragments were ligated onto the *Nde*I/*Xba*I operated pSET152AKE [16] and constructed the *ctd14* overexpression plasmid pCQUT-1268-14. Then, this plasmid was transformed into *Streptomyces parvus* 1268 to generate exconjugants by *Escherichia coli* ET12567/pUZ8002 mediated conjugation. Target exconjugants were subsequently selected on ABB13 plates supplied with 35 μg/mL apramycin to confirm their antibiotic resistance. Then, single colonies were patched onto ABB13 plates containing 35 μg/mL apramycin, and then the correct phenotype (AprR) candidate transformants were further verified by PCR and sequencing.

### 3.6. HPLC Analysis of the Extracts of Wild-Type and Transformant

After fermentation in a modified AM3 medium for 7 days, the cultures of wild-type and transformant of *Streptomyces parvus* 1268 were extracted with ethyl acetate. After the evaporation of ethyl acetate under reduced pressure, the extracts were dissolved in 1.5 mL acetonitrile and analyzed by HPLC at 264 nm with a Cosmosil 5C4-AR-300 Packed column (250 × 4.6 mm, 5 µm) eluting with a linear gradient elution system of CH_3_CN/H_2_O (0–20 min 15:85–90:10; 20.1–25 min 100:0; 25.1–30 min 15:85) at a flow rate of 1 mL/min, on an equipment of Thermo Scientific UltiMate 3000 (ThermoFisher, USA).

### 3.7. Extraction and Isolation

After the fermentation, the liquid and medium were separated by centrifugation. Then, both of them were extracted with equal ethyl acetate three times to afford crude extracts after the solvent evaporation. The residues were combined and subsequently subjected to silica gel CC to obtain twelve fractions (Fr. A1–Fr. A12) using a gradient elution of CH_2_Cl_2_/MeOH (100:0, 99:1, 98:2, 97:3, 96:4, 95:5, 94:6, 93:7, 92:8, 91:9, 90:10, 85:15, *v*/*v*). Fr. A3 was purified by MPLC with an ODS column eluting with MeOH/H_2_O (0:100, 10:90, 20:80, 30:70, 40:60, 50:50, 60:40, 70:30, 80:20, 90:10, 100:0, *v*/*v*) get Fr. B1–Fr. B11. Fr. B7 was further purified with semi-preparative HPLC equipment with a YMC-Pack ODS-A column (250 × 10 mm, 5 μm) eluting with a mixture of CH_3_CN/H_2_O (0–30 min, 45:55–50:50; *v*/*v*) at a flow rate of 3 mL/min to yield compounds **1** (12.7 mg), **2** (7.4 mg) and **3** (8.2 mg) at the retention time of 24.3 min, 17.1 min and 16.1 min, respectively. Fr. B8 was also purified by semi-preparative HPLC using an elution system of CH_3_CN/H_2_O (0–15 min, 75:25–80:20; *v*/*v*) at a flow rate of 3 mL/min to obtain compounds **4** (6.4 mg) and **5** (4.1 mg) at the retention time of 9.2 min and 11.4 min, respectively.

#### 3.7.1. Carpatamide I (**1**)

Light-yellow power; UV (MeOH) λmax (log ε) 272 (4.03), 315 (3.56) nm; ^1^H NMR (400 MHz, CD_3_OD/DMSO-*_d_*_6_) and ^13^C NMR (100 MHz, CD_3_OD/DMSO-*_d_*_6_) data, Table 1, Table 2 and Table S2; ESI-HRMS *m*/*z* 339.1682 [M + Na]^+^ (calcd for C_18_H_24_N_2_O_3_Na 339.1679).

#### 3.7.2. Carpatamide J (**2**)

Light-yellow power; UV (MeOH) λmax (log ε) 272 (4.06), 315 (3.43) nm; ^1^H NMR (400 MHz, CD_3_OD) and ^13^C NMR (100 MHz, CD_3_OD) data, Table 1 and Table 2; ESI-HRMS *m*/*z* 325.1521 [M + Na]^+^ (calcd for C_17_H_22_N_2_O_3_Na 325.1523).

#### 3.7.3. Carpatamide K (**3**)

Light-yellow power; UV (MeOH) λmax (log ε) 272 (4.18), 315 (3.77) nm; ^1^H NMR (400 MHz, CD_3_OD) and ^13^C NMR (100 MHz, CD_3_OD) data, Table 1 and Table 2; ESI-HRMS *m*/*z* 325.1534 [M + Na]^+^ (calcd for C_17_H_22_N_2_O_3_Na, 325.1523).

#### 3.7.4. Carpatamide L (**4**)

Light-yellow power; UV (MeOH) λmax (log ε) 288 (4.02), 315 (3.74 nm; ^1^H NMR (400 MHz, CD_3_OD) and ^13^C NMR (100 MHz, CD_3_OD) data, Table 1 and Table 2; ESI-HRMS *m*/*z* 337.1527 [M + Na]^+^ (calcd for C_18_H_22_N_2_O_3_Na, 337.1523).

#### 3.7.5. Carpatamide M (**5**)

Light-yellow power; UV (MeOH) λmax (log ε) 272 (4.14), 315 (3.64) nm; ^1^H NMR (500 MHz, CD_3_OD) and ^13^C NMR (125 MHz, CD_3_OD) data, Table 1 and Table 2; ESI-HRMS *m*/*z* 353.1843 [M + Na]^+^ (calcd for C_19_H_26_N_2_O_3_Na, 353.1836).

### 3.8. Anti-Inflammatory Activity

The isolates were evaluated for their anti-inflammatory activities by testing the inhibition of NO production in lipopolysaccharide (LPS)-induced RAW 246.7 mouse macrophages following the procedure of the literature [17]. Briefly, the cultured cells were firstly diluted to a density of 5 × 10^5^ cells/well and transferred to new cells to incubate for another day. Then, dexamethasone (TEX 15 μM) and the isolates were added to the corresponding cells. After incubation for 1 day, the plate was detected by Griess reagent for its NO production. Then, the cells were measured at 540 nm for their absorbance.

### 3.9. Cytotoxic Assay

Compounds were tested for their cytotoxic activity against cell lines of A549, HepG2, and HT-29. Cisplatin serves as the positive control. The brief procedure is as follows: cells were first seeded in 96-well plates, then they were harvested and digested with pancreatin. After this, an appropriate concentration of digested cells was seeded to each cell and incubated for 1 day, and then the tested compounds were added to DMSO at appropriate concentrations. After another 24 h at the condition of 37 °C and 5% CO_2_, they were evaluated for their cytotoxic activities using a CCK-8 kit and measured by a microplate reader at 450 nm.

### 3.10. Gene Expression Analysis of ctd14

Total RNA of wild type and transformant strains which were harvested from different media of M-ISP_4_ (1% soluble starch, 0.1% K_2_HPO_4_, 0.1% MgSO_4_.7H_2_O, 0.1% NaCl, 0.2% (NH_4_)_2_SO_4_, 0.2% CaCO_3_, 1 mL trace salt, 3% sea salt, pH 7–7.4), SFM (2% mannitol, 2% soya flour, 3% sea salt) and AM3 after fermentation for 7 d, were extracted using the SV total RNA purification Kit (Promega, Madison, WI, USA) and digested by DNase I (Takara). First-strand cDNA synthesis was accomplished using Invitrogen’s SuperScriptTM Kit (Invitrogen, Waltham, MA, USA), and second-step PCR was carried out under the following conditions: 94 °C for 5 min, 25 cycles of denaturation (94 °C for 25 s), annealing (50 °C for 20 s), and extension (72 °C for 45 s), and a single extension at 72 °C for 5 min. A negative control was accordingly performed in the absence of a template to check for DNA contamination after the DNase I digestion required for RNA purification. Quantitative real-time reverse transcription PCR (qPCR) was performed using the MaximaTM SYBR Green qPCR Mix (MBI) (Shanghai Lianmai Bioengineering Co., Ltd., Shanghai, China) and Applied Biosystem’s 7500 Fast Real-time PCR system (Applied Biosystems, Waltham, MA, USA). 16S rDNA was used as the internal control. The sequence of primers used to analyze the *ctd14* (EPctd14-Fr/Re) and 16S rDNA (EP16S-Fr/Re) were listed in Table S4.

## 4. Conclusions

In conclusion, we isolated a new carpatamide derivative of carpatamide I and found its biosynthetic cluster *ctd*, which is characterized as a 3,4-AHBA synthase, in the genome of *Streptomyces parvus* 1268. Genome analysis identified the positive transcriptional regulatory gene of *ctd14* in cluster *ctd*. Overexpression of Ctd 14 helped us to obtain four new carpatamide derivatives of carpatamides J–M and to improve the fermentation titer of carpatamide I simultaneously. However, bioactivity results showed that they displayed weaker anti-inflammatory activity and cytotoxicity against cell lines of A549, HT-29, and HepG2 than that of carpatamides A and C.

## Figures and Tables

**Table 1:** ^1^H NMR spectroscopic data (400/500 MHz) for compounds **1**–**5** in CD_3_OD.

Position	*δ*_H_, Multi. (*J* in Hz)				
	Carpatamide I (1) ^1^	Carpatamide J (2) ^1^	Carpatamide K (3) ^1^	Carpatamide L (4) ^1^	Carpatamide M (5) ^2^
1					
2					
3	7.52, d (2.0)	7.51, d (2.0)	7.52, d (2.0)	8.13, d (2.4)	7.51, d (2.0)
4					
5	6.89, dd (8.4, 2.0)	6.89, dd (8.4, 2.0)	6.89, dd (8.4, 2.0)	7.22, dd (8.4, 2.4)	6.89, dd (8.0, 2.0)
6	6.79, d (8.4)	6.79, d (8.4)	6.79, d (8.4)	6.89, d (8.4)	6.79, d (8.0)
7	2.83, t (7.2)	2.83, t (7.2)	2.83, t (7.2)	7.47, d (15.6)	2.83, t (7.5)
8	2.47, t (7.2)	2.47, t (7.2)	2.47, t (7.2)	6.46, d (15.6)	2.47, t (7.5)
9					
10					
11	6.22, d (15.2)	6.20, d (15.2)	6.22, d (15.2)	6.24, d (15.6)	6.20, d (15.0)
12	7.28, dd (15.2, 10.4)	7.28, dd (15.2, 10.4)	7.28, dd (15.0, 10.4)	7.30, dd (15.2, 10.4)	7.27, dd (15.0, 10.5)
13	6.28, dd (15.2, 10.4)	6.29, dd (15.2, 10.4)	6.26, dd (15.2, 10.4)	6.29, dd (15.2, 10.4)	6.30, dd (15.0, 10.5)
14	6.16, dd (15.2, 7.2)	6.19, dd (15.2, 6.8)	6.15, dd (15.2, 6.8)	6.17, dd (14.8, 7.2)	6.18, dd (15.0, 7.5)
15	2.10, t (7.2)	2.19, q (7.2)	2.45, m	2.10, t (7.2)	2.22, q (7.5)
16	1.73, m	1.49, dt (7.2)	1.07, d (6.8)	1.73, m	1.35, q (7.5)
17	0.94, d (6.7)	0.95, t (7.2)	1.07, d (6.8)	0.94, d (6.8)	1.59, m
18	0.94, d (6.7)			0.94, d (6.8)	0.93, d (7.0)
19					0.93, d (7.0)

^1^ 400 MHz, ^2^ 500 MHz.

**Table 2:** ^13^C NMR spectroscopic data (100/125 MHz) for compounds **1**–**5** in CD_3_OD.

Position	Carpatamide I (1) ^1^	Carpatamide J (2) ^1^	Carpatamide K (3) ^1^	Carpatamide L (4) ^1^	Carpatamide M(5) ^2^
1	148.1, C	148.0, C	147.9, C	151.8, C	148.1, C
2	127.4, C	127.2, C	127.2, C	128.2, C	127.2, C
3	123.5, CH	123.4, CH	123.4, CH	122.4, CH	123.4, CH
4	133.7, C	133.5, C	133.5, C	127.7, C	133.5, C
5	126.9, CH	126.7, CH	126.7, CH	127.2, CH	126.7, CH
6	117.7, CH	117.6, CH	117.6, CH	117.3, CH	117.6, CH
7	32.3, CH_2_	32.2, CH_2_	32.2, CH_2_	143.0, CH	32.2, CH_2_
8	38.7, CH_2_	38.6, CH_2_	38.6, CH_2_	118.6, CH	38.6, CH_2_
9	178.4, C	178.3, C	178.3, C	171.6, C	178.3, C
10	167.7, C	167.6, C	167.6, C	167.7, C	167.6, C
11	123.0, CH	122.8, CH	122.9, CH	123.1, CH	122.7, CH
12	143.9, CH	143.8, CH	144.1, CH	144.0, CH	143.9, CH
13	131.1, CH	130.0, CH	127.0, CH	131.1, CH	129.7, CH
14	144.1, CH	144.9, CH	151.7, CH	144.2, CH	145.3, CH
15	43.5, CH_2_	36.1, CH_2_	32.8, CH	43.5, CH_2_	32.0, CH_2_
16	29.7, CH	23.1, CH_2_	22.3, CH_3_	29.7, CH	39.2, CH
17	22.9, CH_3_	14.0, CH_3_	22.3, CH_3_	22.9, CH_3_	28.8, CH
18	22.9, CH_3_			22.9, CH_3_	22.8, CH_3_
19					22.8, CH_3_

^1^ 100 MHz, ^2^ 125 MHz.

**Table 3:** Anti-inflammatory and cytotoxic activity of compounds **1**–**5**.

Compounds	Inflammatory Inhibition Rate (%)	Cell Lines (IC_50_ μM)		
		A549	HT-29	HepG2
Carpatamide I (**1**)	17.37	45.17 ± 2.01	32.17 ± 1.24	35.22 ± 1.98
Carpatamide J (**2**)	13.84	42.15 ± 1.04	37.19 ± 1.11	42.11 ± 0.21
Carpatamide K (**3**)	11.45	41.51 ± 1.95	31.15 ± 2.02	29.33 ± 2.14
Carpatamide L (**4**)	1.22	39.37 ± 0.99	38.45 ± 0.99	25.12 ± 0.98
Carpatamide M (**5**)	20.48	35.47 ± 1.01	22.59 ± 1.55	31.42 ± 1.44
Cisplatin	69.32	1.53 ± 0.44	3.55 ± 0.31	1.39 ± 0.21

## Data Availability

Data are contained within the article or Supplementary Materials.

## Supplementary Materials

The following supporting information can be downloaded at: https://www.mdpi.com/article/10.3390/md22110521/s1, the spectral NMR data of **1**–**5**, Cytotoxic activity data, 1D (^1^H NMR, ^13^CNMR) spectra, 2D (COSY, HSQC and HMBC) spectra, and ESI-HRMS of compounds **1**–**5**.
