# Supplementary material for: Genome-Based Mining of Carpatamides I–M and Their Candidate Biosynthetic Gene Cluster
Source: Mar Drugs. 2024 Nov 20;22(11):521. doi: 10.3390/md22110521 (PMC11595529; doi:10.3390/md22110521)
Supplement: Supplementary file 1 [file marinedrugs-22-00521-s001.zip › marinedrugs-3314147-supplementary.pdf]

Supplementary Material

Genome-Based Mining of Carpatamides I–M and Their Candidate Biosynthetic Gene Cluster

Shu-Mei Shen[1,†], Yun-Chang Xie[2,†], Li-Rong Tu[1], Miao-Er Wu[2], Yan-Min Wang[2], Chun-Hui Song[2] and Yu-Hui Sun[3,*], Ming-He Luo[1,*]

[1] School of Pharmacy and Bioengineering, Chongqing University of Technology, Chongqing, 400054, China; shenm51627@stu.cqut.edu.cn (S.-M.S.); tlr110825@stu.cqut.edu.cn (L.-R.T.)

[2]College of Life Sciences, Jiangxi Normal University, Jiangxi, 330022, China; xieyunchang@jxnu.edu.cn (Y.-C.X.); miaokanshijie@163.com (M.-E.W.); wym0826333@163.com (Y.-M.W.); schxzx1977@126.com (C.-H.S.)

[3] School of Pharmacy, Huazhong University of Science and Technology, Wuhan 430030, China;

[*] Correspondence: yhsun@whu.edu.cn (Y.-H.S.); lmh353083@126.com (M.-H.L.); Tel.: +86-027-68756642 (Y.-H.S.); +86-13677697348 (M.-H.L.)

[†] These authors contributed equally.

Content

Table S1. Deduced functions of ORFs in the *ctd* cluster. 4-6

Table S2. 1D NMR spectral data (400 MHz) for compound **1** in DMSO-*d6*....................6

Table S3. Bacterial strains and plasmids used in this study. 7

Table S4. List of oligonucleotide primers used in this study 7

Figure S1. The homologous comparison between *ctd* and identified BGCs 8

Figure S2. ESI-HRMS spectrum for carpatamide I **(1)** 9

Figure S3. The ^{1}H NMR (400 MHz) spectrum of carpatamide I **(1)** in CD_3OD. 9

Figure S4. The ^{13}C NMR (100 MHz) spectrum of carpatamide I **(1)** in CD_3OD. 10

Figure S5. The COSY spectrum of carpatamide I **(1)** in CD_3OD. 10

Figure S6. The HSQC spectrum of carpatamide I **(1)** in CD_3OD. 11

Figure S7. The HMBC spectrum of carpatamide I **(1)** in CD_3OD. 11

Figure S8. The ^{1}H NMR (400 MHz) spectrum of carpatamide I **(1)** in DMSO-*d6*. 9

Figure S9. The ^{13}C NMR (100 MHz) spectrum of carpatamide I **(1)** in DMSO-*d6*. 10

Figure S10. The COSY spectrum of carpatamide I **(1)** in DMSO-*d6*. 10

Figure S11. The HSQC spectrum of carpatamide I **(1)** in DMSO-*d6*.. 11

Figure S12. The HMBC spectrum of carpatamide I **(1)** in DMSO-*d6*.. 11

Figure S13. Scheme presentation of *ctd14* overexpression and verification. 15

Figure S14. ESI-HRMS spectrum for carpatamide J **(2)** 16

Figure S15. The ^{1}H NMR (400 MHz) spectrum of carpatamide J **(2)** in CD_3OD. 16

Figure S16. The ^{13}C NMR (100 MHz) spectrum of carpatamide J **(2)** in CD_3OD. 17

Figure S17. The ^{1}H-^{1}H COSY spectrum of carpatamide J **(2)** in CD_3OD. 17

Figure S18. The HSQC spectrum of carpatamide J **(2)** in CD_3OD. 17

Figure S19. The HMBC spectrum of carpatamide J **(2)** in CD_3OD. 18

Figure S20. The NOESY spectrum of carpatamide J **(2)**) in CD_3OD. 19

Figure S21. ESI-HRMS spectrum for carpatamide K **(3)** 19

Figure S22. The ^{1}H NMR (400 MHz) spectrum of carpatamide K **(3)** in CD_3OD. 20

Figure S23. The ^{13}C NMR (100 MHz) spectrum of carpatamide K **(3)** in CD_3OD. 20

Figure S24. The ^{1}H-^{1}H COSY spectrum of carpatamide K **(3)** in CD_3OD. 21
Figure S25. The HSQC spectrum of carpatamide K **(3)** in CD_3OD. 21
Figure S26. The HMBC spectrum of carpatamide K **(3)** in CD_3OD. 22
Figure S27. ESI-MS spectrum for carpatamide L **(4)** 22
Figure S28. ESI-HRMS spectrum for carpatamide L **(4)** 22
Figure S29. The ^{1}H NMR (400 MHz) spectrum of carpatamide L **(4)** in CD_3OD. 23
Figure S30. The ^{13}C NMR (100 MHz) spectrum of carpatamide L **(4)** in CD_3OD. 23
Figure S31. The ^{1}H-^{1}H COSY spectrum of carpatamide L **(4)** in CD_3OD. 24
Figure S32. The HSQC spectrum of carpatamide L **(4)** in CD_3OD. 24
Figure S33. The HMBC spectrum of carpatamide L **(4)** in CD_3OD. 25
Figure S34. The NOESY spectrum of carpatamide L **(4)** in CD_3OD. 25
Figure S35. ESI-HRMS spectrum for carpatamide M **(5)** 26
Figure S36. The ^{1}H NMR (400 MHz) spectrum of carpatamide M **(5)** in CD_3OD. 26
Figure S37. The ^{13}C NMR (100 MHz) spectrum of carpatamide M **(5)** in CD_3OD. 27
Figure S38. The ^{1}H-^{1}H COSY spectrum of carpatamide M **(5)** in CD_3OD. 27
Figure S39. The HSQC spectrum of carpatamide M **(5)** in CD_3OD. 28
Figure S40. The HMBC spectrum of carpatamide M **(5)** in CD_3OD. 28
Figure S41. Phylogenetic analysis of the *Streptomyces parvus* 1268. 29
Reference .... Error! Bookmark not defined.

Table S1. Deduced functions of ORFs in the *ctd* cluster.

ORF	Size	Proposed function	ID/SM	Protein homologue and origin
1	58	Homoserine kinase	32/48	(Q0AHY7.1) *Nitrosomonas eutropha* C91
2	482	Amino-acid carrier protein AlsT	50/68	(Q45068.1) *Bacillus subtilis subsp. subtilis* str. 168
3	212	RNA polymerase sigma factor HrpL	30/49	(P37929.1) *Pseudomonas syringae* pv. syringae
4	179	Pentatricopeptide repeat containing protein At4g16390	32/45	(Q8GWE0.3) *Arabidopsis thaliana*
5	314	2-oxo-3-(phosphooxy) propyl 3-oxoalkanoate synthase	43/58	(O24738.1) *Streptomyces virginiae*
6	222	A-factor receptor protein	38/53	(Q9ZN78.1) *Streptomyces griseus*
7	348	2-alkyl-3-oxoalkanoate reductase	30/44	(Q8EG63.1) *hewanella oneidensis MR-1*
8	234	A-factor receptor protein	29/48	(Q9ZN78.1) *Streptomyces griseus* MR-1
9	321	Probable transcriptional regulatory protein Meso_3192	40/57	(Q11DG1.1) *Chelativorans* sp. BNC1
10	397	4-hydroxyprotoasukamycin monooxygenase	58/71	(D7P5X0.1) *Streptomyces nodosus subsp. asukaensis*
11	414	Acyltransferase MdmB	40/52	(Q00718.1) *Streptomyces mycarofaciens*
12	615	Asparagine synthetase	43/59	(O05272.3) *Bacillus subtilis subsp. subtilis* str. 168
13	406	Uncharacterized MFS-type transporter YbcL	32/56	(O34663.1) *Bacillus subtilis subsp. subtilis* str. 168
14	195	response regulator transcription factor	99/99	(WP_217689959.1) *Streptomyces* sp. CAI 127
15	276	4'-phosphopantetheinyl transferase	29/47	(P39144.1) *Bacillus subtilis*
16	293	Arylamine N-acetyltransferase / N-hydroxyarylamine O-acetyltransferase	37/46	(Q00267.1) *Salmonella enterica subsp. enterica serovar Typhimurium* str. LT2
17	172	Short=3-hydroxyl-ACP dehydratase FERN	28/46	(A0A3Q7HWE4.1) *Solanum lycopersicum*

18	153	UPF0336 protein SAV_4901	33/52	(Q82DR8.1) *Streptomyces avermitilis* MA-4680
19	366	3-methyl-2-oxobutanoate dehydrogenase subunit alpha	45/58	(P9WIS3.1) *Mycobacterium tuberculosis* H37Rv
20	326	Pyruvate dehydrogenase E1 component subunit beta	65/78	(Q6ABX8.1) *Leifsonia xyli* subsp. xyli str. CTCB07
21	422	Dihydrolipoyllysine-residue acetyltransferase component of pyruvate dehydrogenase complex	51/63	(Q6ABX9.1) *Leifsonia xyli* subsp. xyli str. CTCB07
22	209	HTH-type transcriptional regulator TtgR	32/48	(Q9AIU0.1) *Pseudomonas putida* DOT-T1E
23	584	2-isopropylmalate synthase	86/92	(Q82BV3.1) *Streptomyces avermitilis* MA-4680
24	83	ACP	39/67	(A0LT89.1) *Acidothermus cellulolyticus* 11B
25	360	Beta-ketoacyl-ACP synthase III 1	55/68	(P72392.1) *Streptomyces coelicolor* A3(2)
26	377	Beta-ketoacyl-ACP synthase III	37/57	(Q0TRH0.1) *Clostridium perfringens* ATCC 13124
27	290	Proansamycin X synthase	27/42	(O52547.1) *Amycolatopsis mediterranei* S699
28	395	Protoasukamycin 4-monooxygenase	30/44	(D7P5V0.1) *Streptomyces nodosus subsp. asukaensis*
29	278	2-amino-4,5-dihydroxy-6-oxo-7-(phosphonooxy)heptanoate synthase	52/70	(B1VTI8.1) *Streptomyces griseus* subsp. griseus NBRC 13350
30	377	3,4-AHBA synthase	52/68	(B1VTI7.1) *Streptomyces griseus* subsp. griseus NBRC 13350
31	488	4-MHA-AMP	48/60	(P80435.3) *Streptomyces anulatus*
32	399	Enoyl-[acyl-carrier-protein] reductase	46/62	(A5FE91.1) *Flavobacterium johnsoniae* UW101
33	201	4-methyl-3-hydroxyanthranilic acid carrier protein	44/63	(D6R237.1) *Streptomyces anulatus*
34	414	3-oxoacyl-[acyl-carrier-protein] synthase 2	32/48	(Q83E37.1) *Coxiella burnetii* RSA 493

35	242	Unknown	-	-
36	170	DHNA-CoA hydrolase	39/55	(P77781.1) *Escherichia coli* K-12
37	187	NADH-dependent FMN reductase AsuE2	54/66	(D7P5W0.1) *Streptomyces nodosus* subsp. asukaensis
38	234	3-oxoacyl-[acyl-carrier-protein] reductase MabA	55/74	(P9WGT3.1) *Mycobacterium tuberculosis* H37Rv
39	202	HTH-type transcriptional regulator TcmR	33/49	(P39885.1) *Streptomyces glaucescens*
40	318	Retinol dehydrogenase 12	39/54	(Q8BYK4.1) *Mus musculus*
41	583	Steroid-24-oyl-CoA synthetase	37/51	(Q0S4D9.1) *Rhodococcus jostii* RHA1

Size: Numbers indicate amino acids;

ID: identity; SM: similarity;

Table S2. 1D NMR spectral data (400 MHz) for compound **1** in DMSO-d_6.

position	δ_C	δ_H, multi. (J in Hz)
1	146.2, C	
2	126.2, C	
3	123.0, CH	7.58, d (2.0)
4	131.9, C	
4-OH		9.80, brs
5	124.6, CH	6.80, dd (8.0, 2.0)
6	116.1, CH	6.76, d (8)
7	30.4, CH_2	2.68, dd (9.6, 7.2)
8	37.1, CH_2	2.29, dd (8.4, 6.0)
9	173.5, C	
10	164.5, C	
11	121.9, CH	6.23, dd (15.2, 10.4)
12	141.0, CH	7.16, dd (14.8, 10.4)
13	129.6, CH	6.36, d (15.2)
14	141.8, CH	6.17, m
15	41.7, CH_2	2.05, t (6.4)
16	27.8, CH	1.68, m
17	22.2, CH_3	0.88, d (6.8)
18	22.2, CH_3	0.88, d (6.8)
19		
2-NH		9.51, s
9-NH_2		7.28, s; 6.73, s

Table S3. Bacterial strains and plasmids used in this study.

Strains/Plasmids	Characteristics	Reference
Escherichia coli		
DH10B	Host for general cloning	Invitrogen
ET12567/pUZ8002	Donor strain for conjugation between *E. coli* and *Streptomyces*	
***Streptomyces* sp.**		
Streptomyces parvus 1268	Wild-type, carpatamides producing strain	This work
1268-Ctd14	overexpression of *ctd14*	This work
Plasmids		
pSET152AKE	*E. coil-Streptomyces* shuttle vector	[1]Xie Y, et al. 2014
pCQUT-1268-14	Recombinant construct for *ctd14* overexpression	This work

Table S4. List of oligonucleotide primers used in this study.

Primers	Oligonucleotide sequences (5' to 3')	Restriction site
Ctd14-up	CAAAGGAGTGTC**CATATG**GTGACCACTCTG CTTCACGAG	*Nde*I
Ctd14-re	GTCGACTCTAGA**TCTAGA**TTAGGAGACCAG TAACCCCTCC	*Xba*I
Ctd14-confirm-up	GCTGCGCCGATGGTTTCTACAAAGATC	
Ctd14-confirm-re	GGCATGGACGAGAAGGACATAGCC	
EPctd14-Fr	CATGGACGAGAAGGACATAGC	
EPctd14-Re	CATGGTGCGGTTGGGACAGTTC	
EP16S-Fr	CATTCGATACGGGCTAGCTAG	
EP16S-Re	CTCCTGTGAGTCCCCATCAC	

Note: The underlined nucleotides indicate introduced restriction sites.

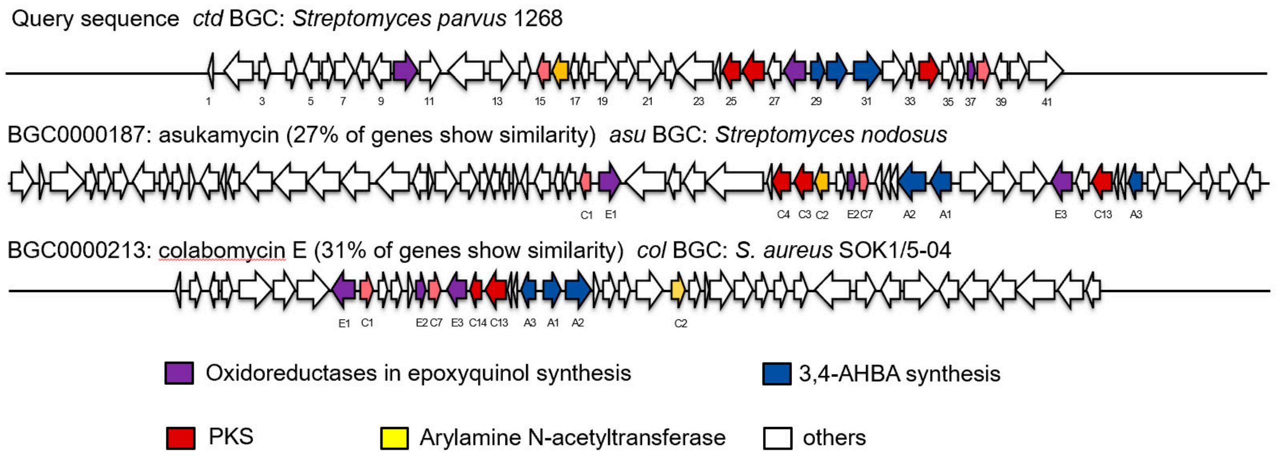

Figure S1. The homologous comparison between *ctd* and identified BGCs including BGC of asukamycin (NCBI GenBank: GQ926890.1) and BGC of colabomycin E (NCBI GenBank: KF850685.1) using AntiSMASH 7.0.

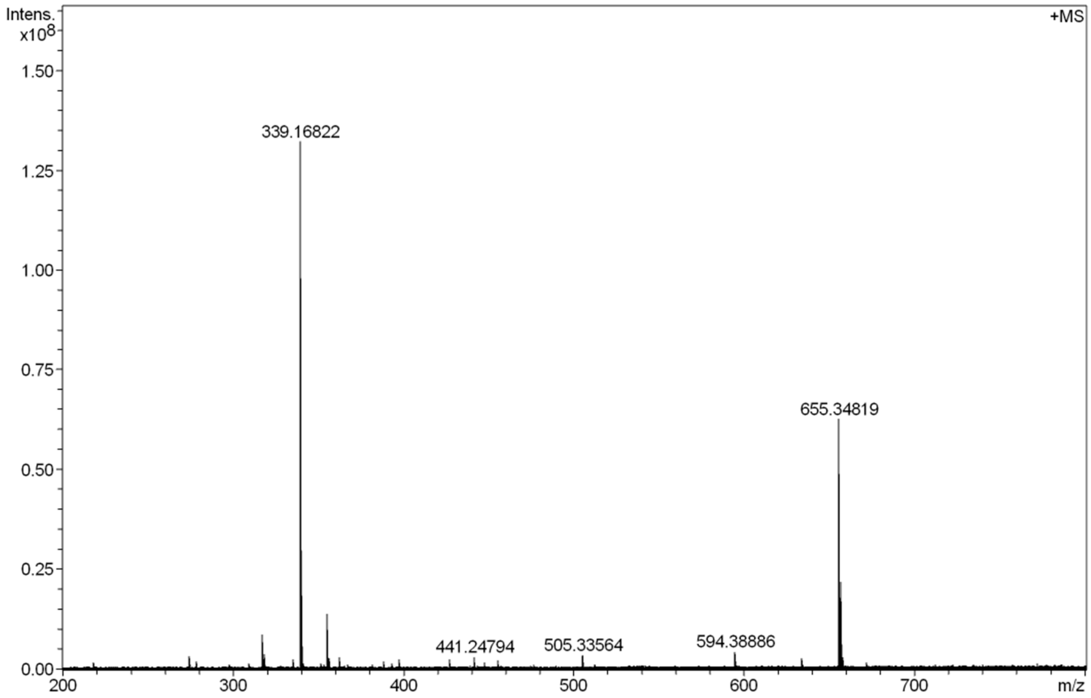

Figure S2. ESI-HRMS spectrum for carpatamide I (**1**)

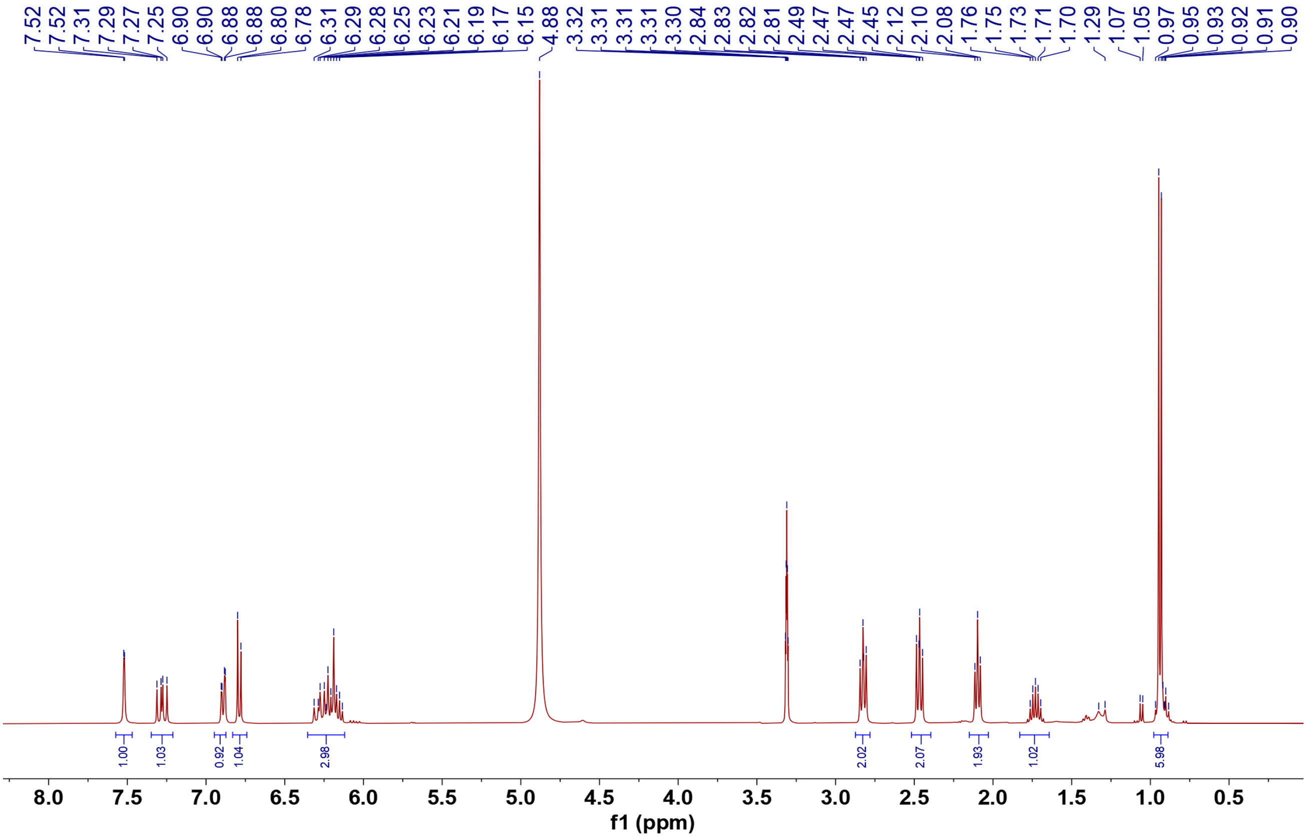

Figure S3. The ^{1}H NMR (400 MHz) spectrum of carpatamide I (**1**) in CD_3OD.

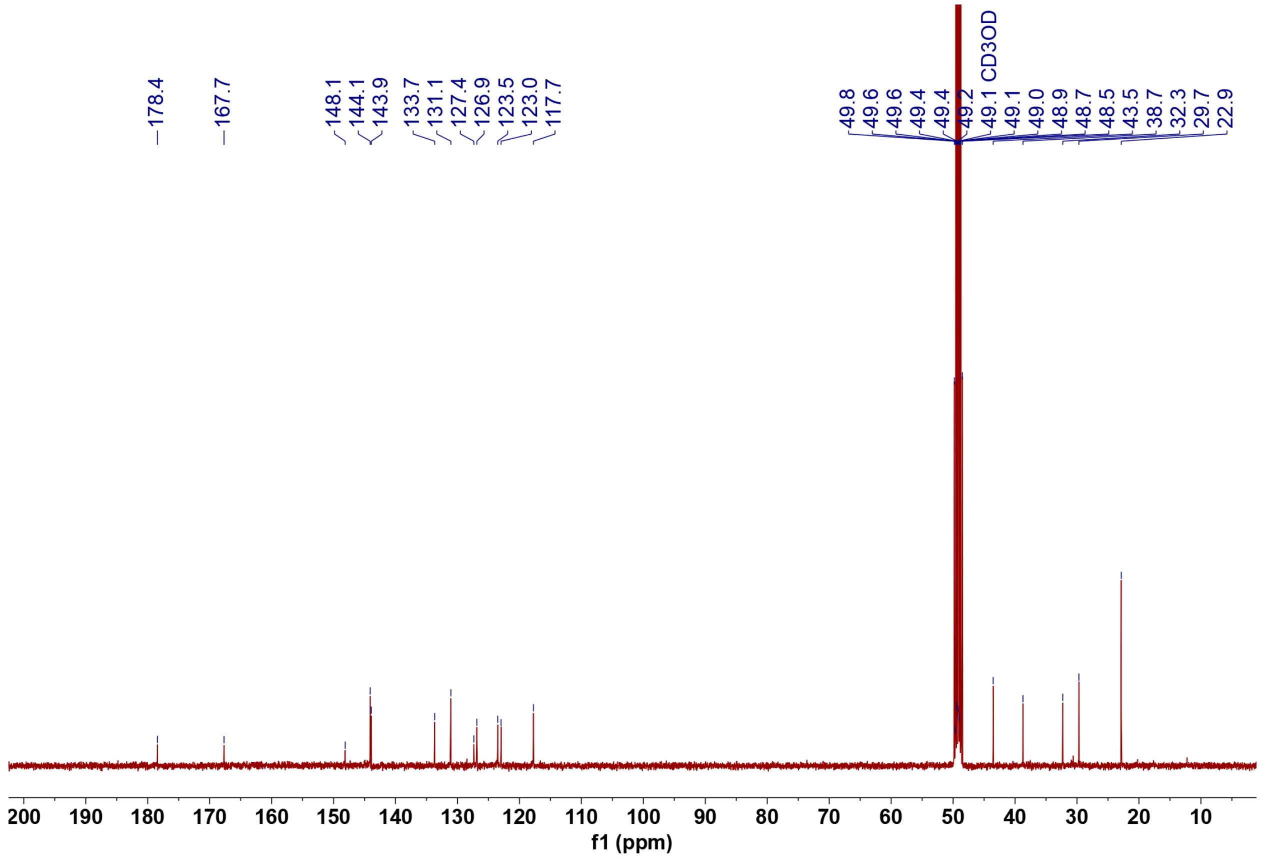

Figure S4. The ^{13}C NMR (100 MHz) spectrum of carpatamide I (**1**) in CD_3OD.

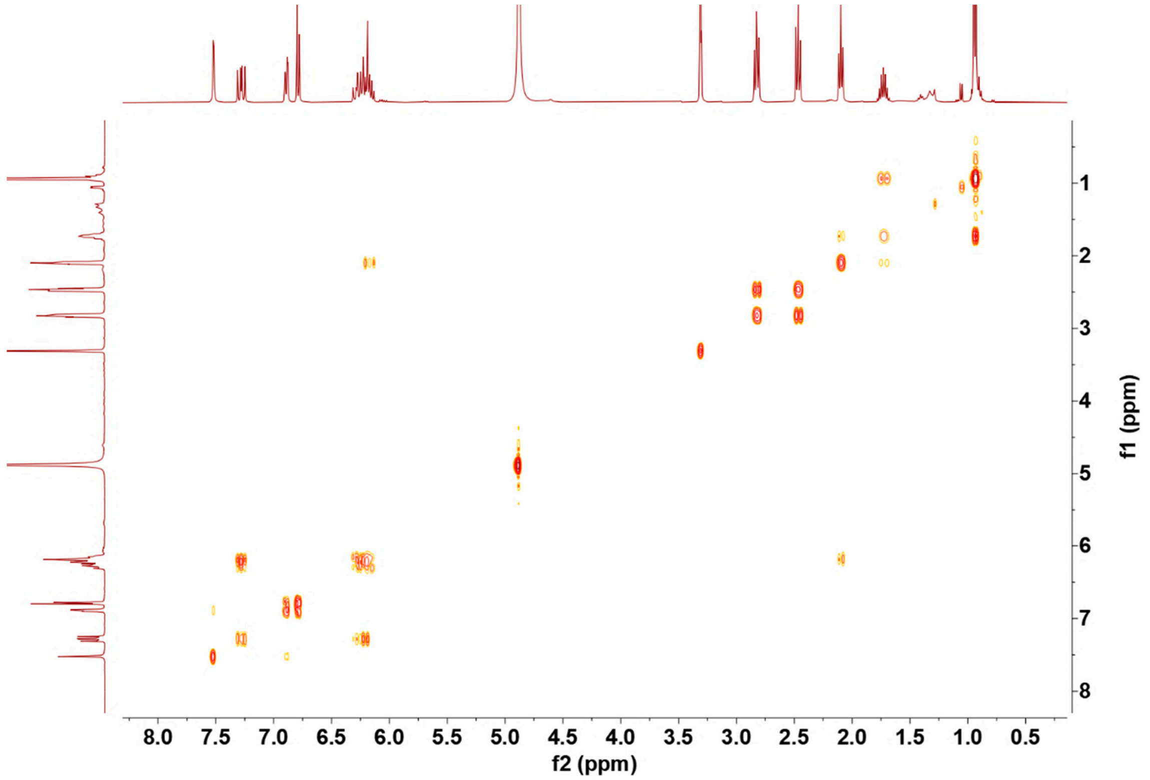

Figure S5. The COSY spectrum of carpatamide I (**1**) in CD_3OD.

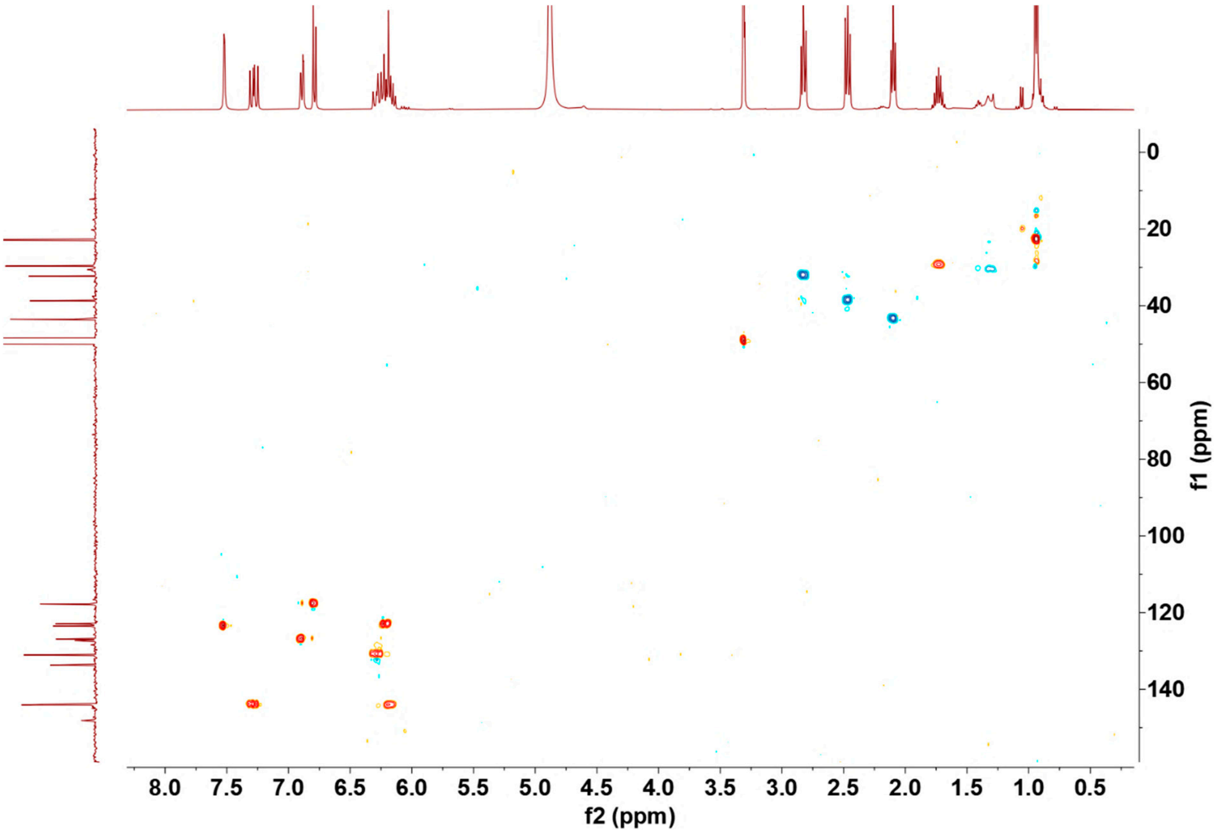

Figure S6. The HSQC spectrum of carpatamide I (**1**) in CD_3OD.

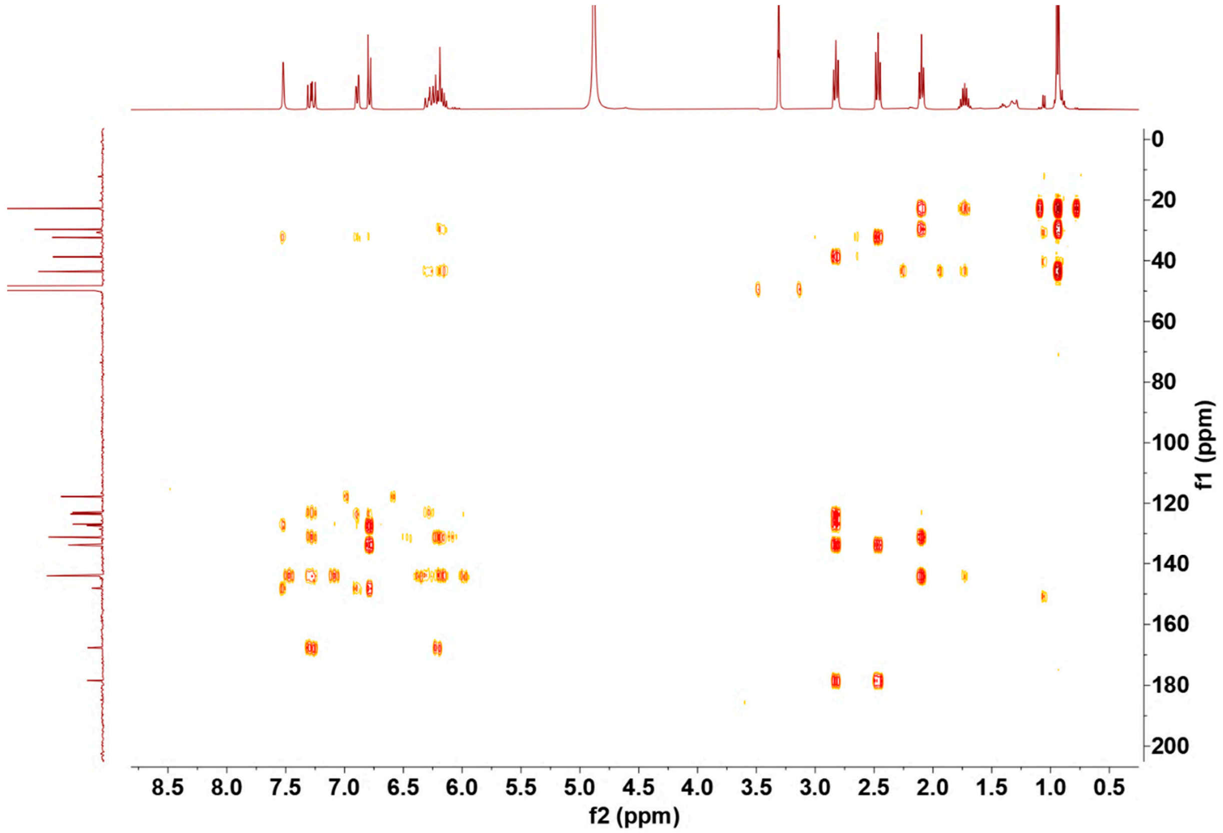

Figure S7. The HMBC spectrum of carpatamide I (**1**) in CD_3OD.

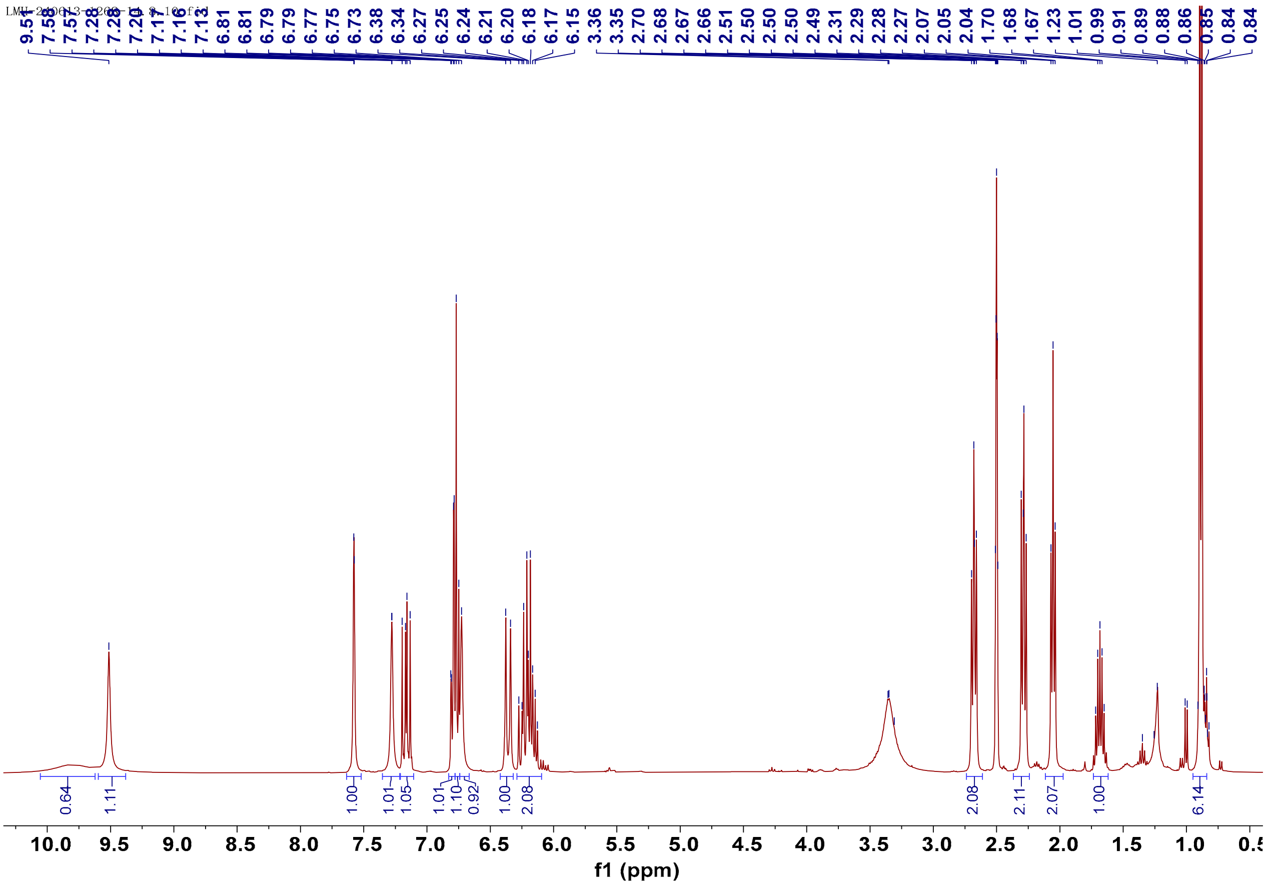

Figure S8. The ^{1}H NMR (400 MHz) spectrum of carpatamide I (1) in DMSO-*d6*.

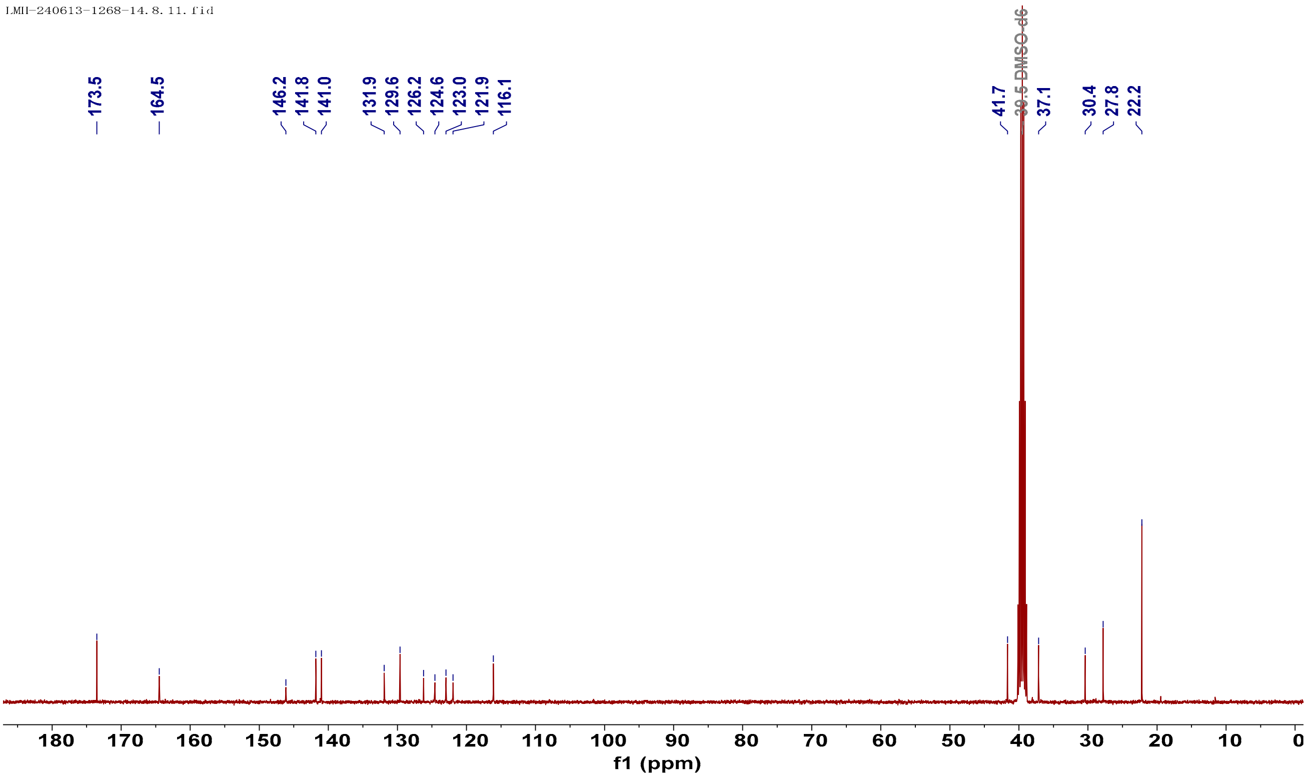

Figure S9. The ^{13}C NMR (100 MHz) spectrum of carpatamide I (1) in DMSO-*d6*.

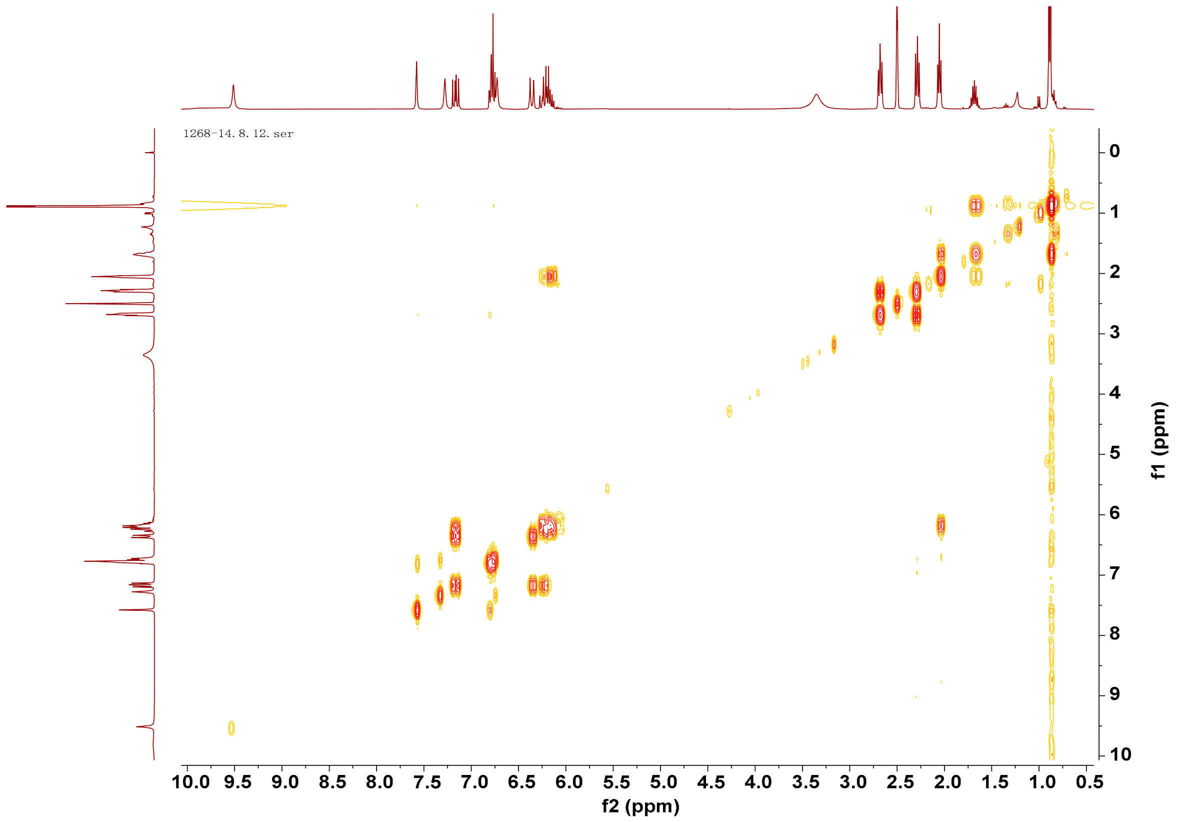

Figure S10. The COSY spectrum of carpatamide I (1) in DMSO-*d6*.

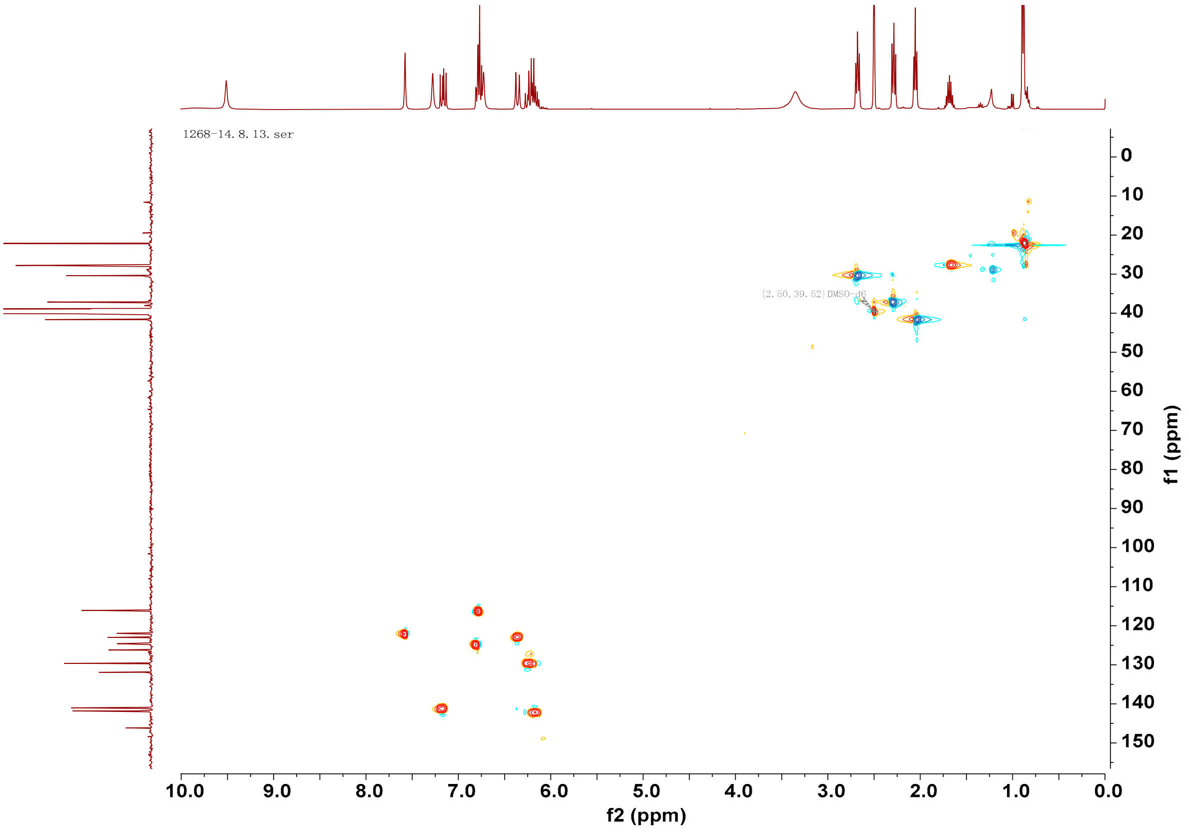

Figure S11. The HSQC spectrum of carpatamide I (1) in DMSO-*d6*.

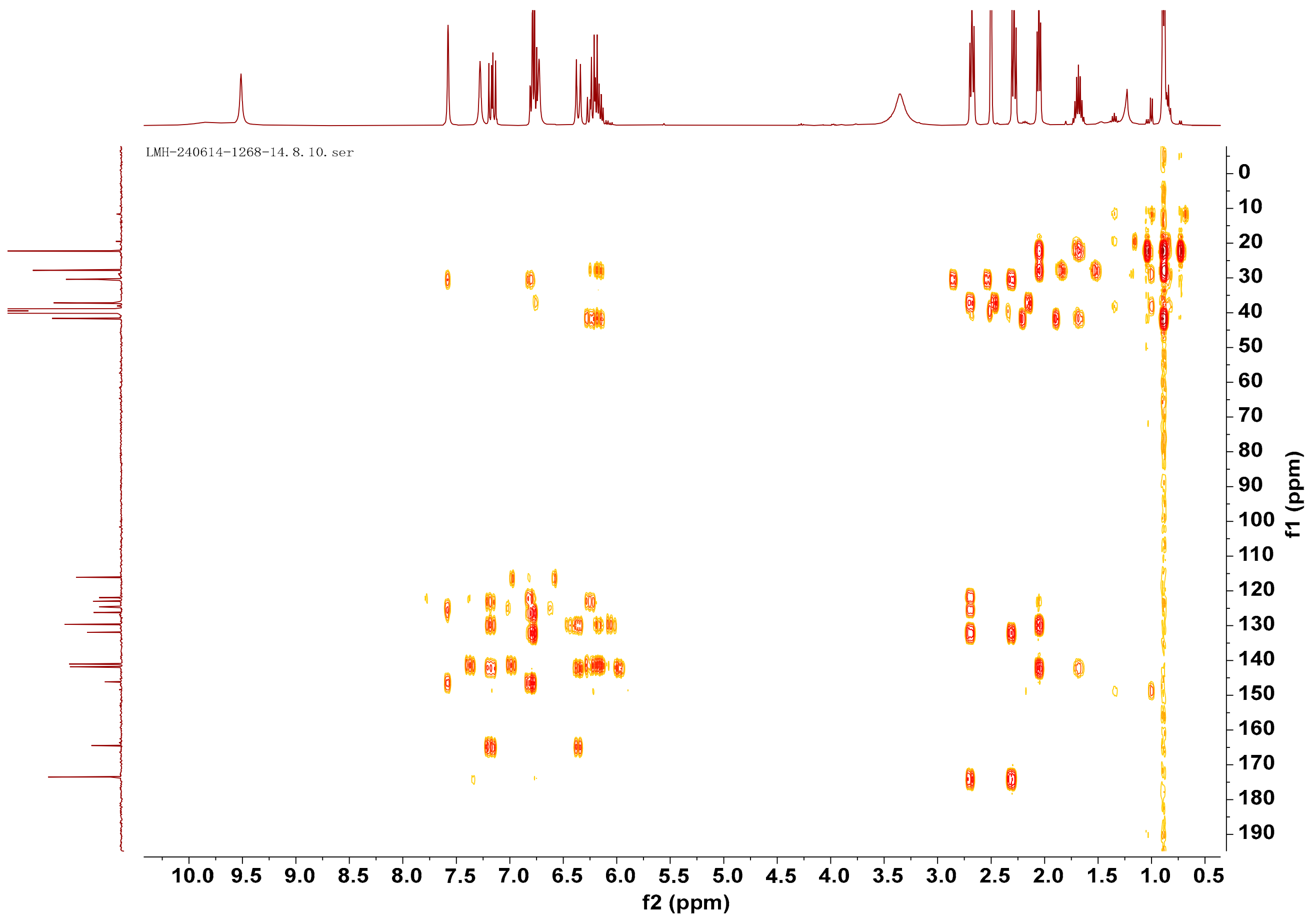

Figure S12. The HMBC spectrum of carpatamide I (1) in DMSO-$_{d6}$.

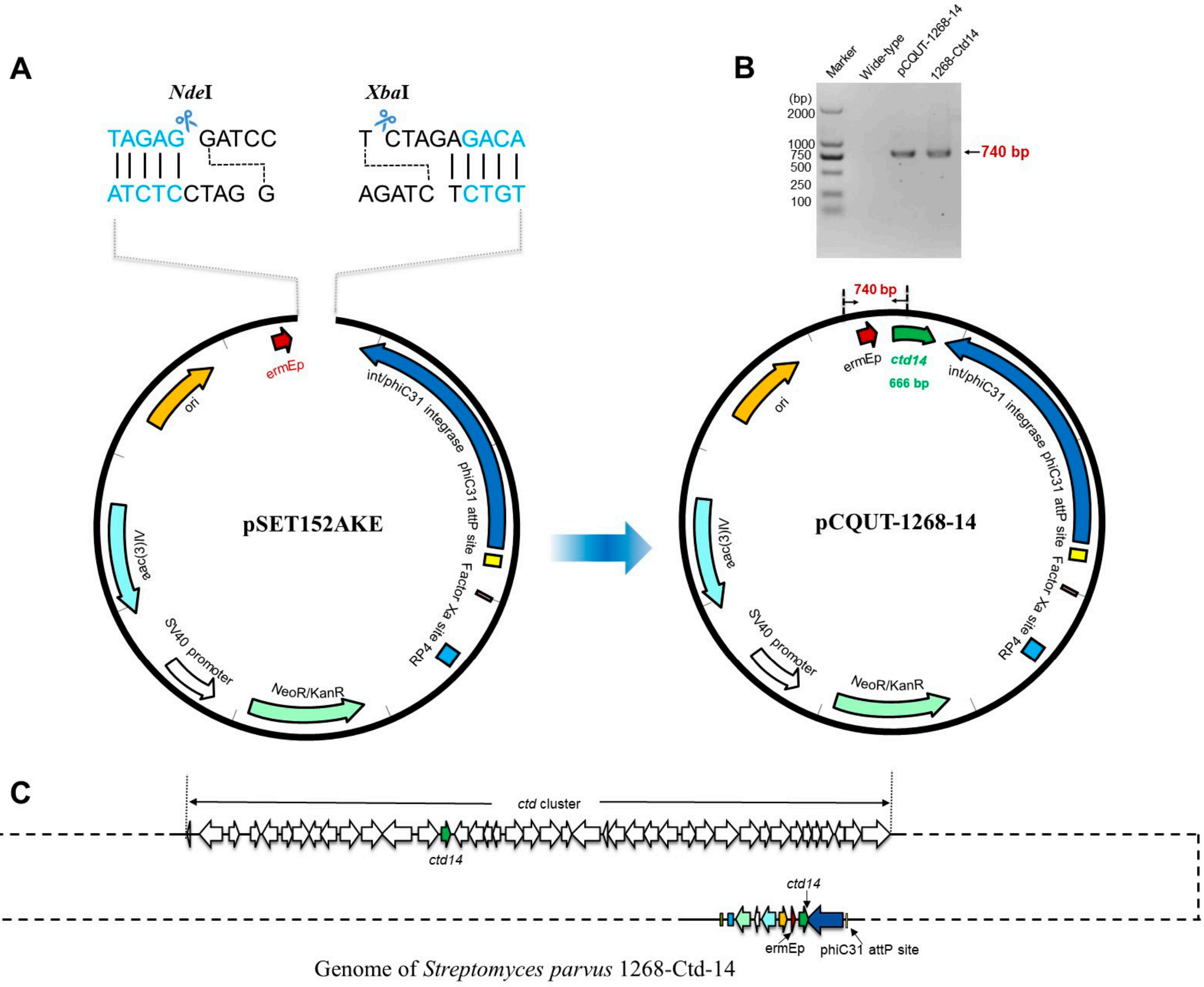

Figure S13. Scheme presentation of *ctd14* overexpression and verification. (A) Construction of recombinant plasmid pCQUT-1268-14 from pSET152AKE. (B) The mutant is confirmed by PCR amplification and sequencing using the genomic DNA from the overexpression mutant of *Streptomyces parvus* 1268-Ctd14 and wild-type (negative control), and plasmid pCQUT-1268-14 (positive control) as template, respectively. (C) Genome of *Streptomyces parvus* 1268-Ctd14.

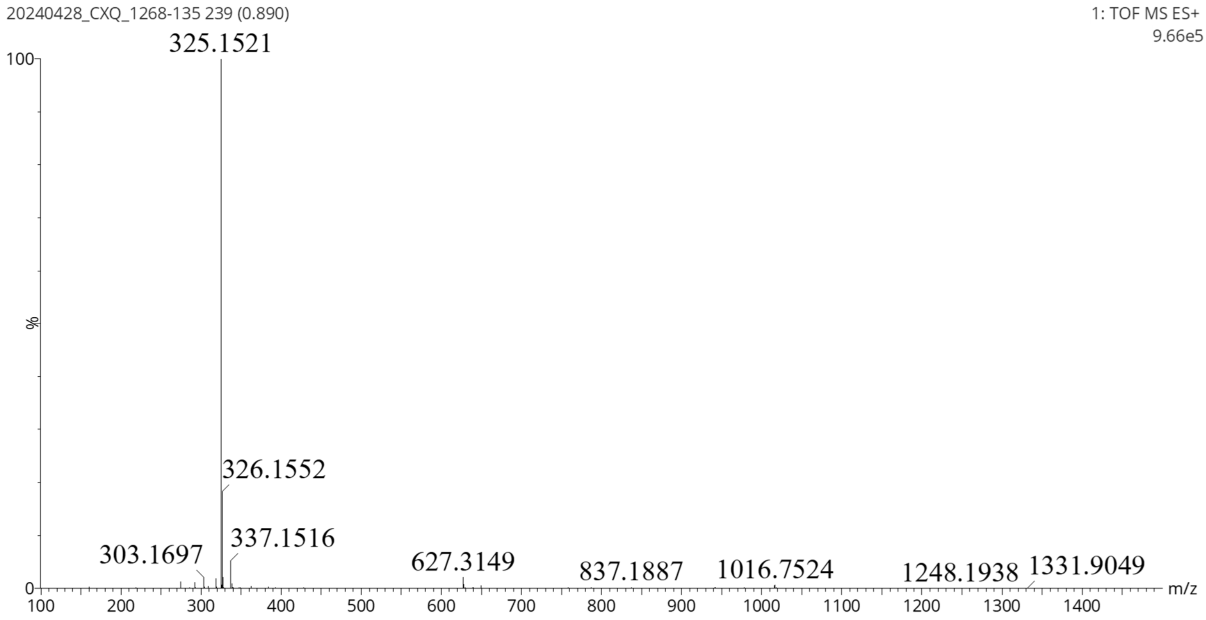

Figure S14. ESI-HRMS spectrum for carpatamide J (**2**)

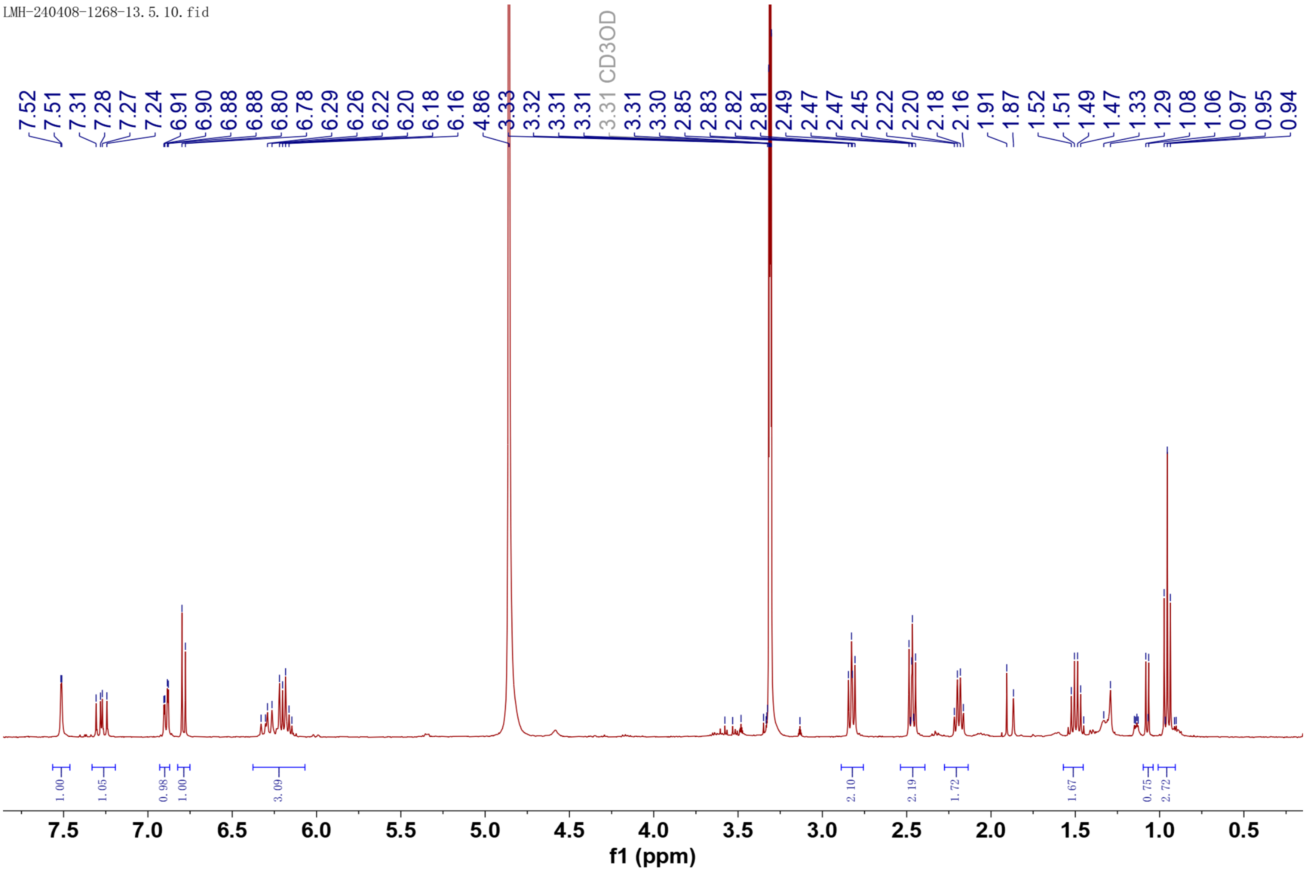

Figure S15. The ^{1}H NMR (400 MHz) spectrum of carpatamide J (**2**) in CD_3OD.

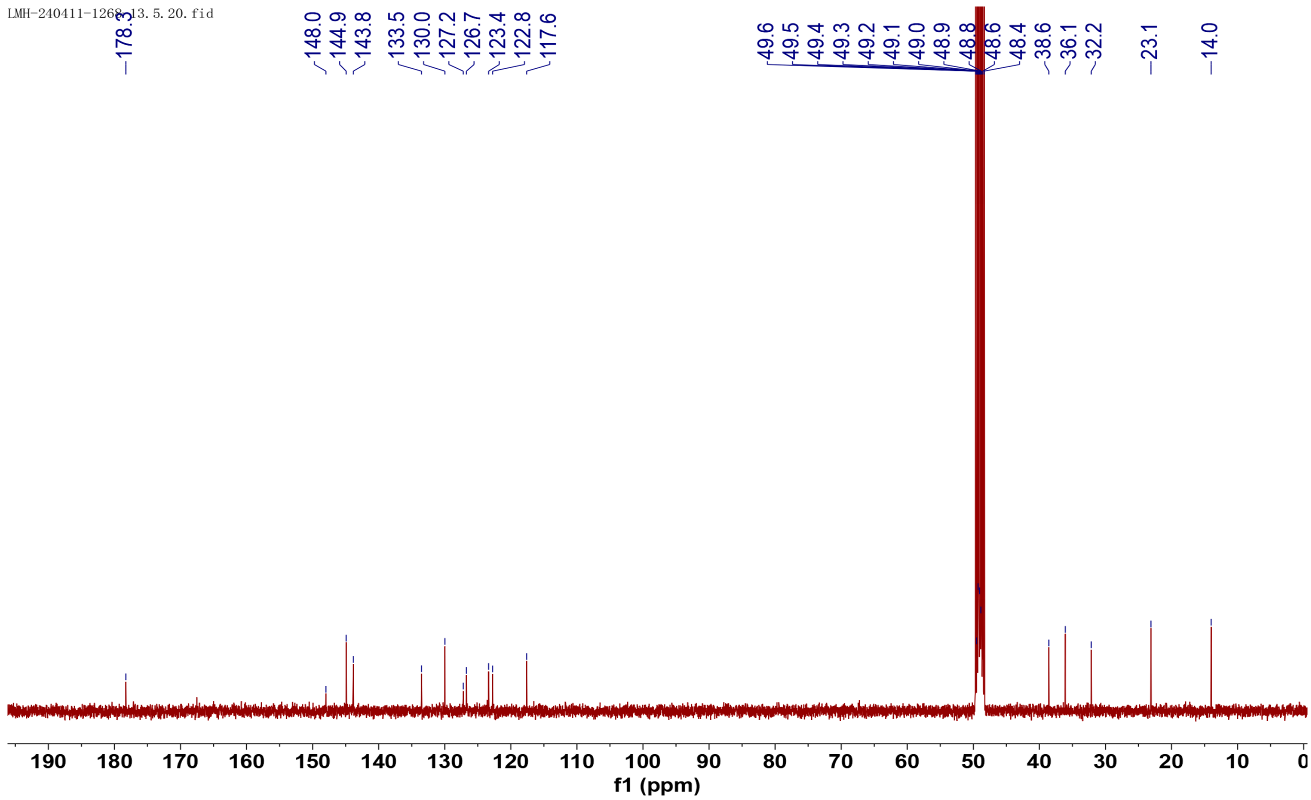

Figure S16. The ^{13}C NMR (100 MHz) spectrum of carpatamide J (**2**) in CD_3OD.

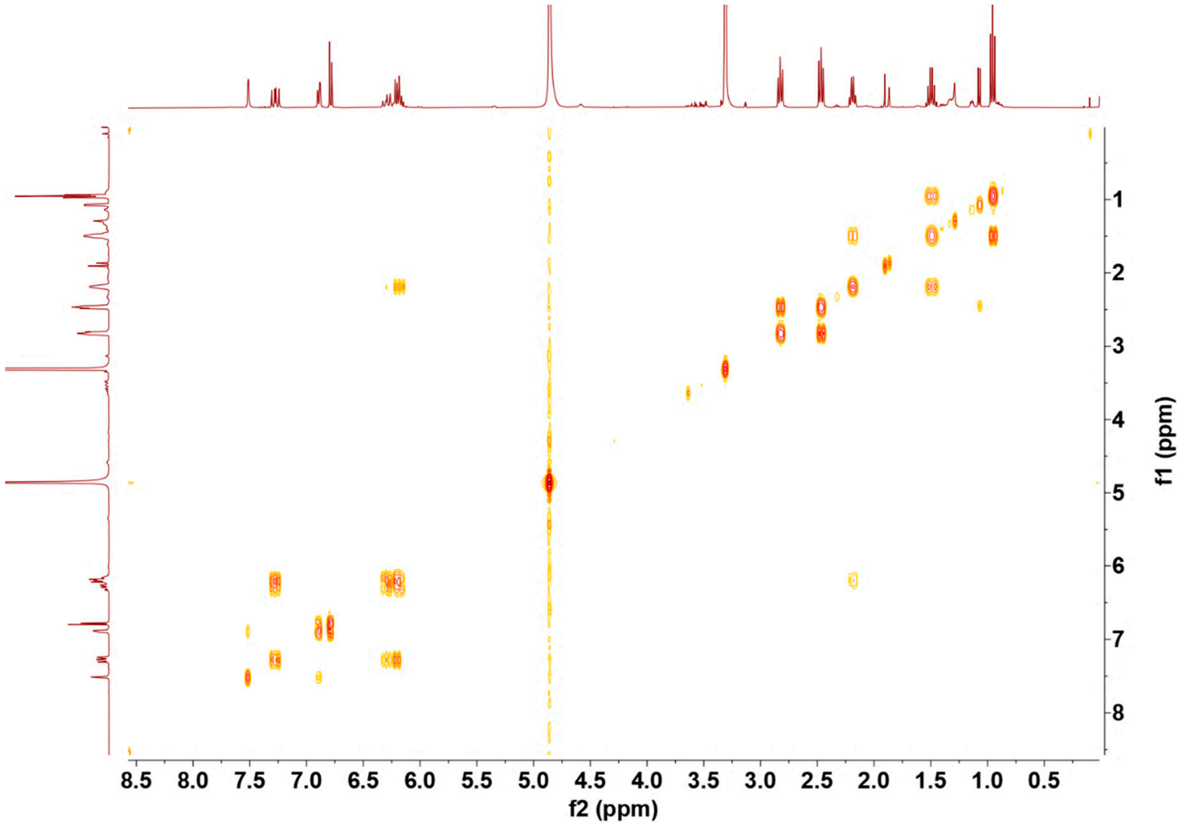

Figure S17. The ^{1}H-^{1}H COSY spectrum of carpatamide J (**2**) in CD_3OD.

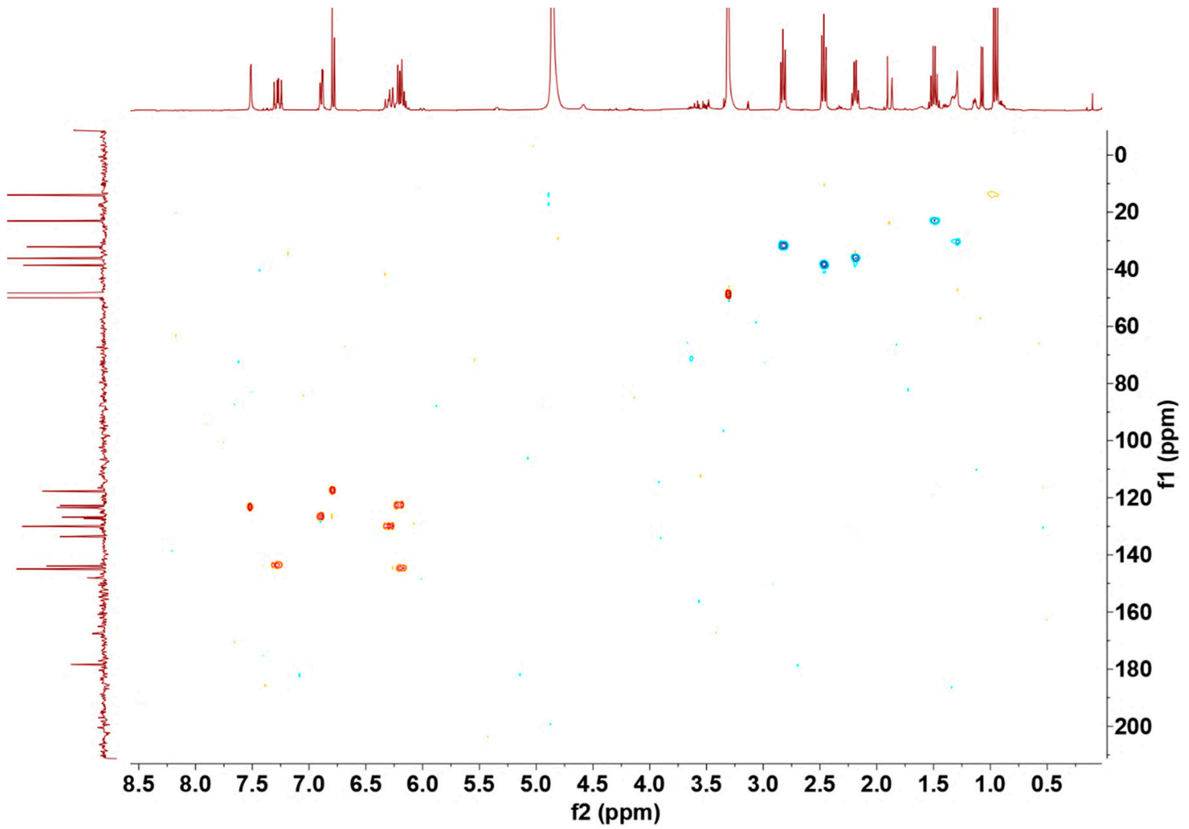

Figure S18. The HSQC spectrum of carpatamide J (**2**) in CD_3OD.

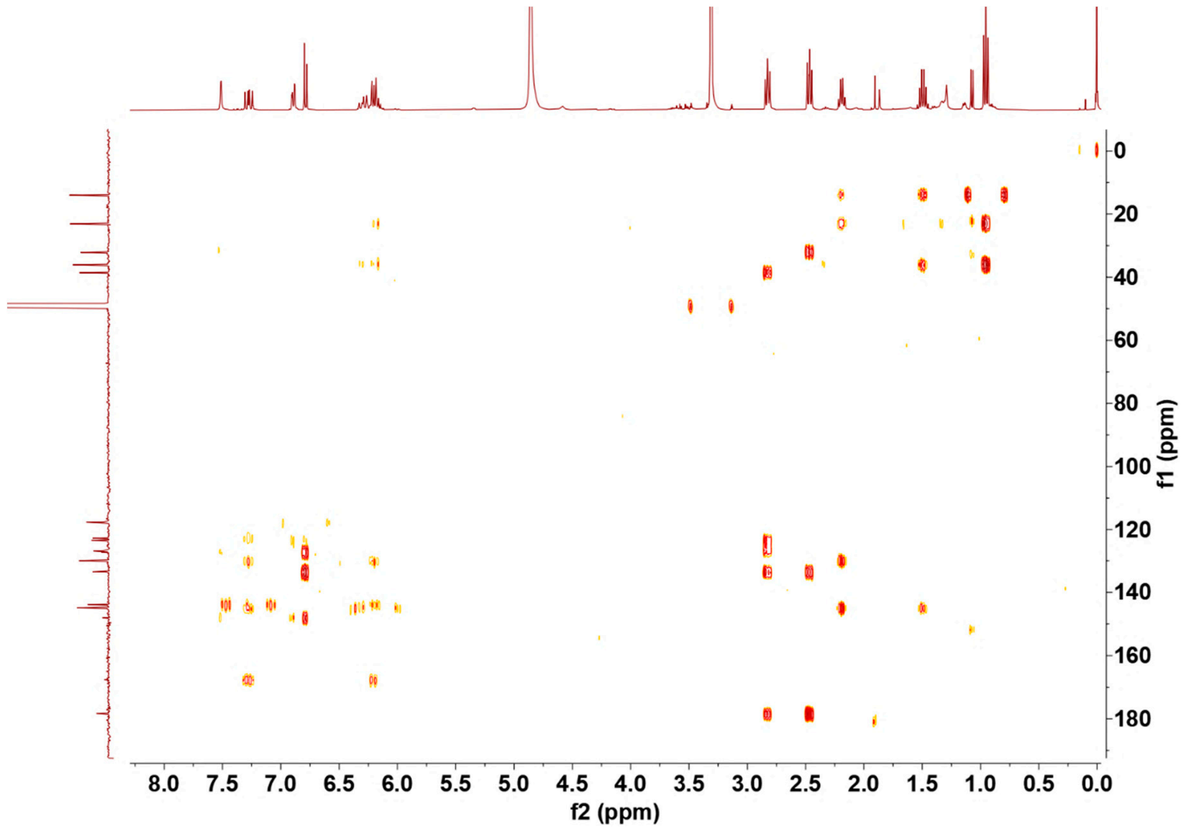

Figure S19. The HMBC spectrum of carpatamide J (**2**) in CD_3OD.

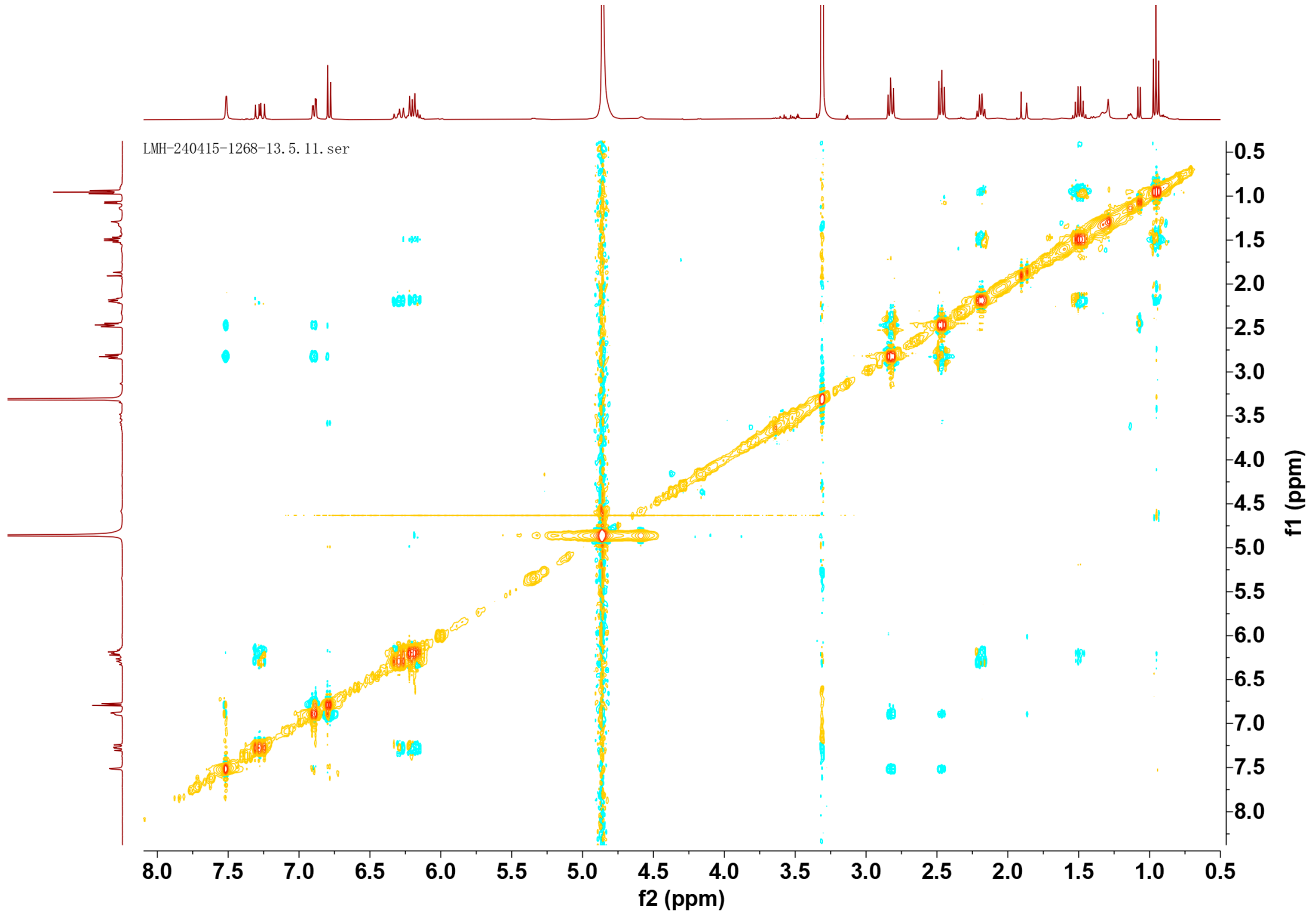

Figure S20. The NOESY spectrum of carpatamide J (**2**)) in CD_3OD.

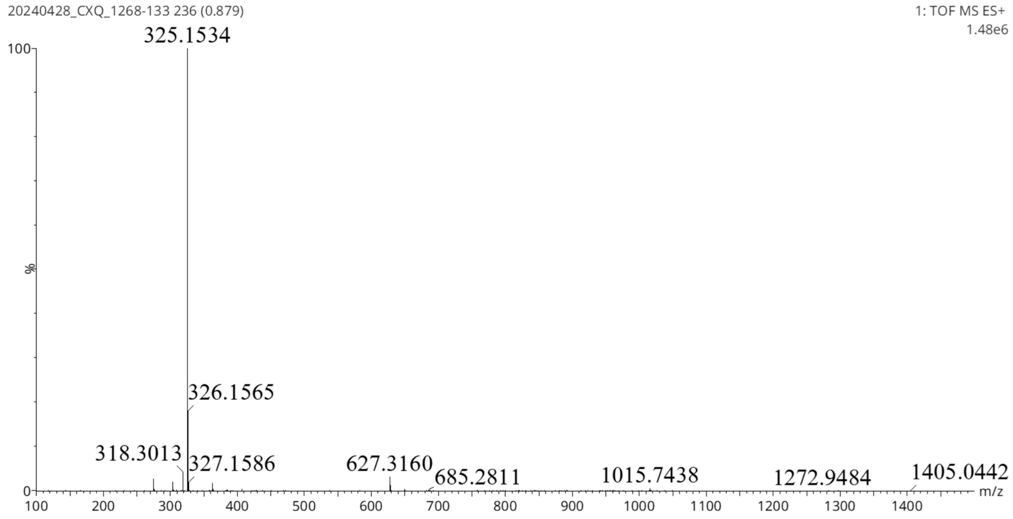

Figure S21. ESI-HRMS spectrum for carpatamide K (**3**)

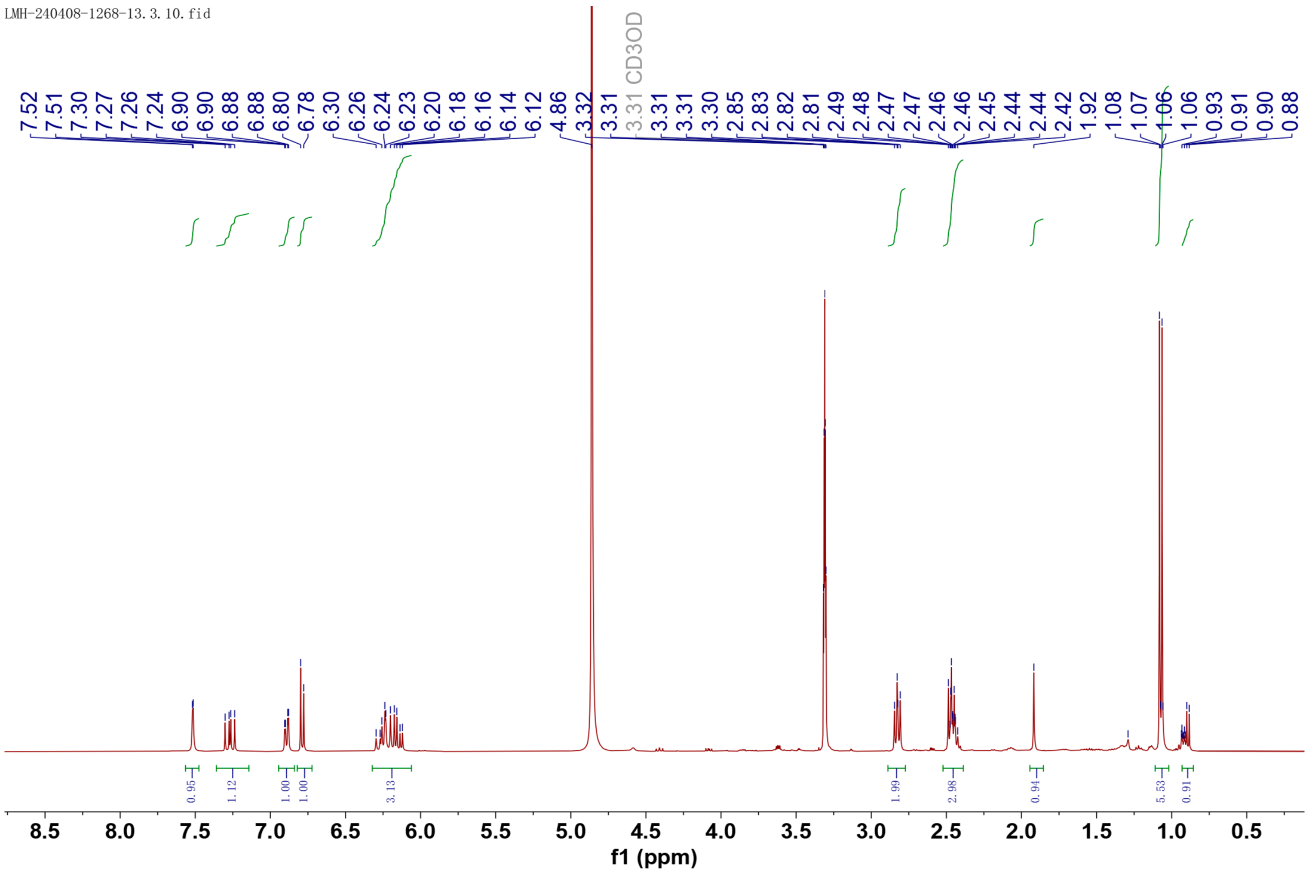

Figure S22. The ^{1}H NMR (400 MHz) spectrum of carpatamide K (**3**) in CD3OD.

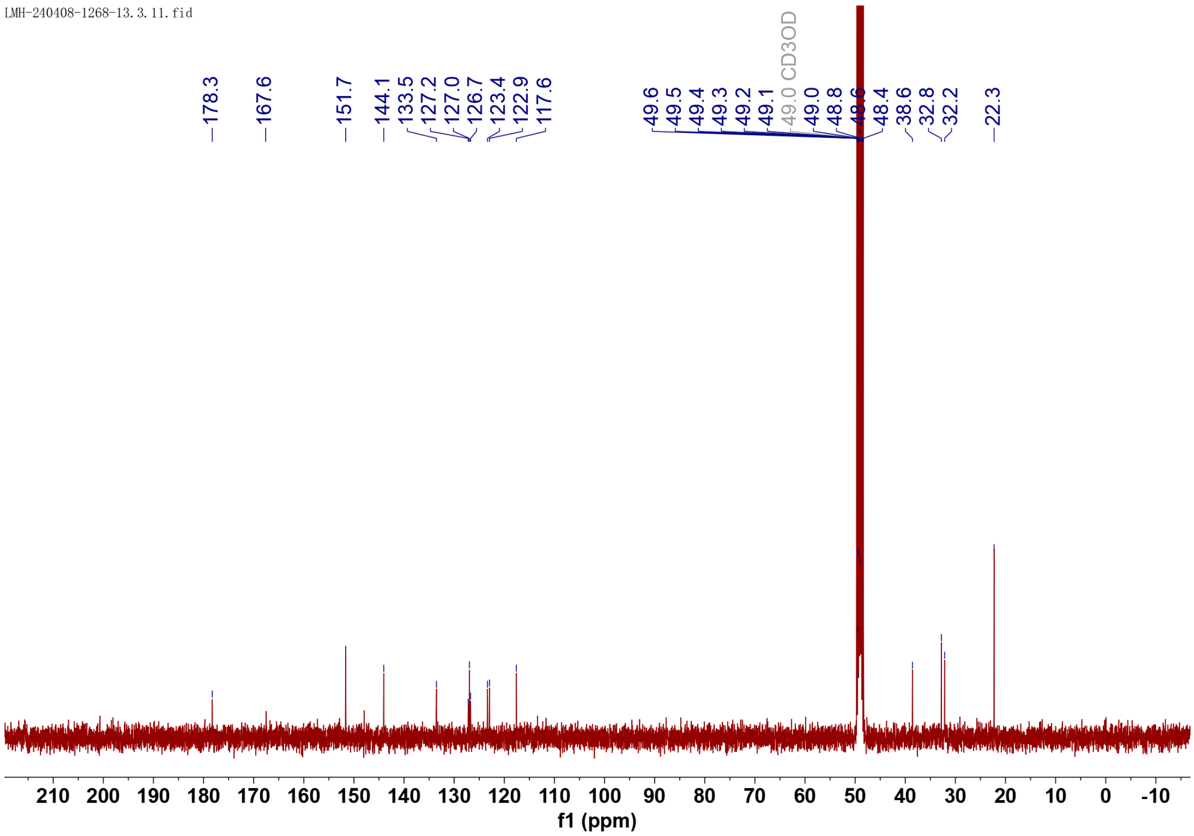

Figure S23. The ^{13}C NMR (100 MHz) spectrum of carpatamide K (**3**) in CD3OD.

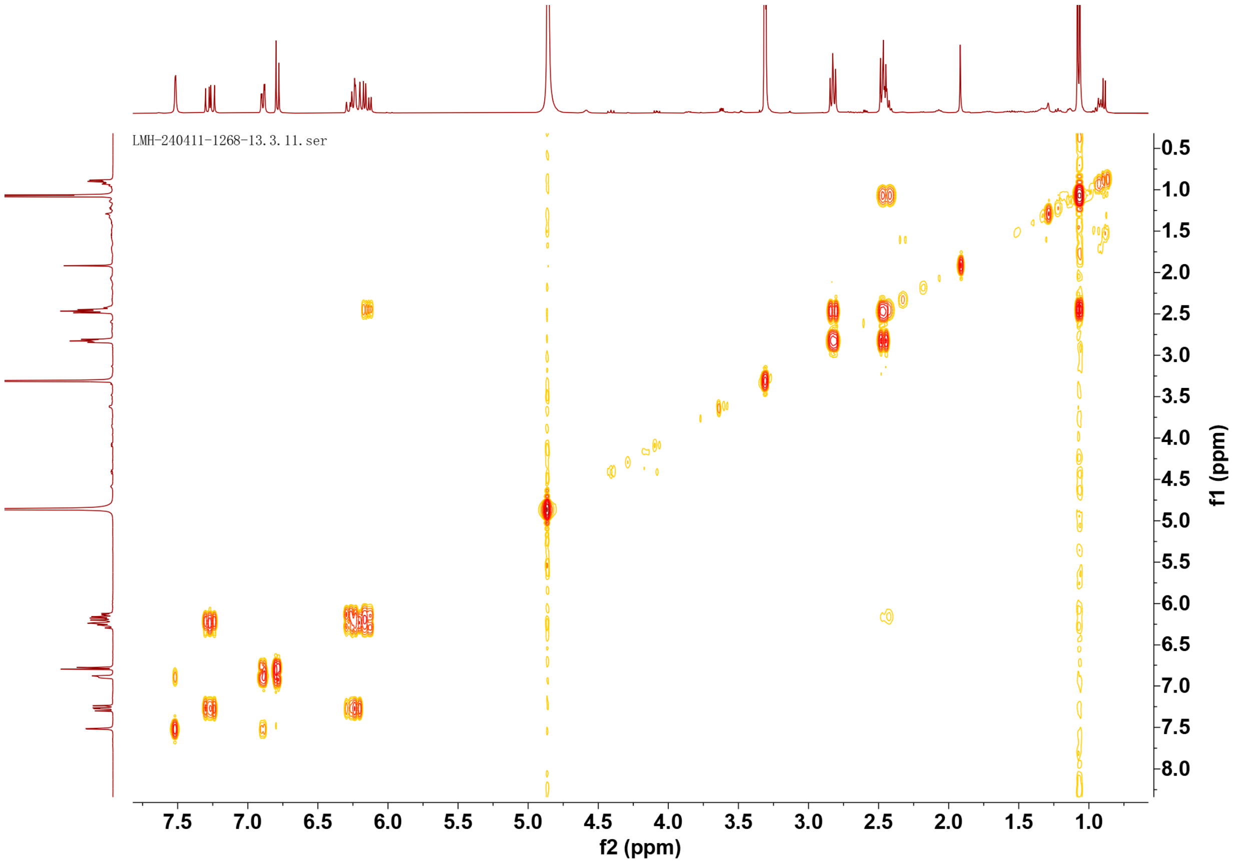

Figure S24. The ^{1}H-^{1}H COSY spectrum of carpatamide K (**3**) in CD_3OD.

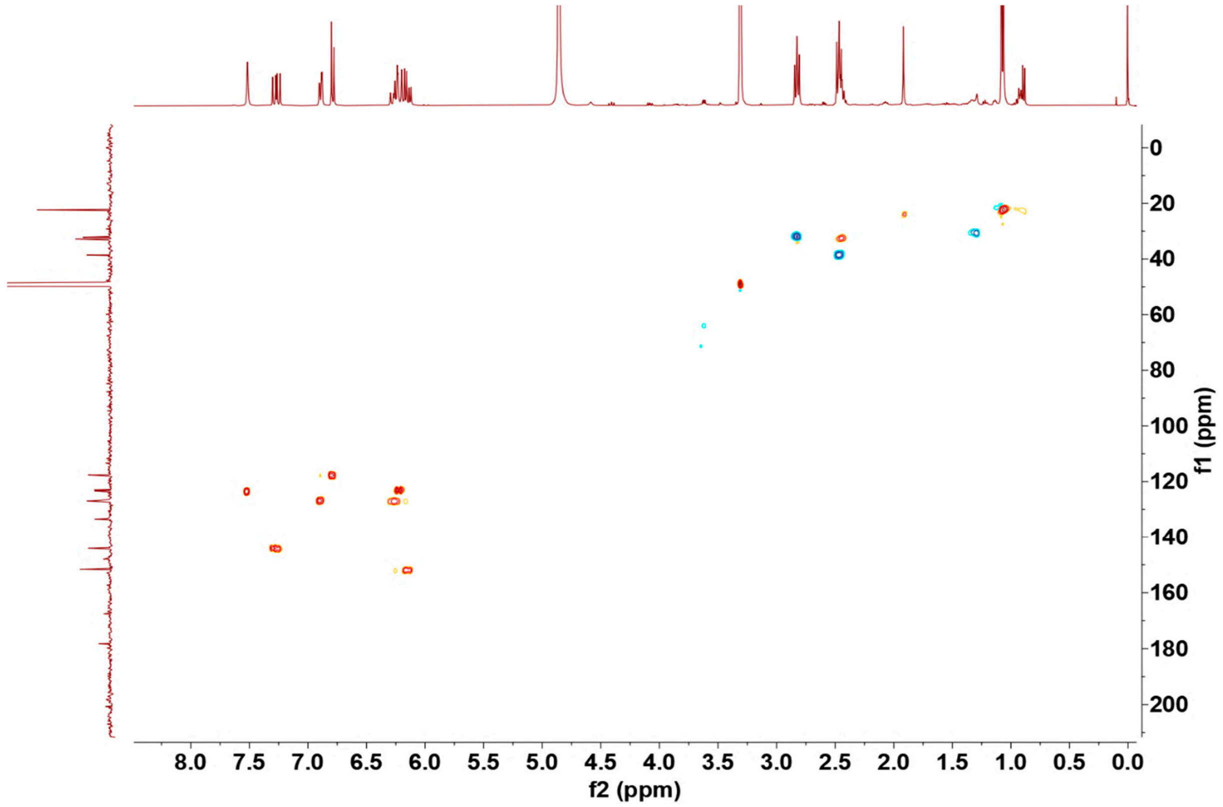

Figure S25. The HSQC spectrum of carpatamide K (**3**) in CD_3OD.

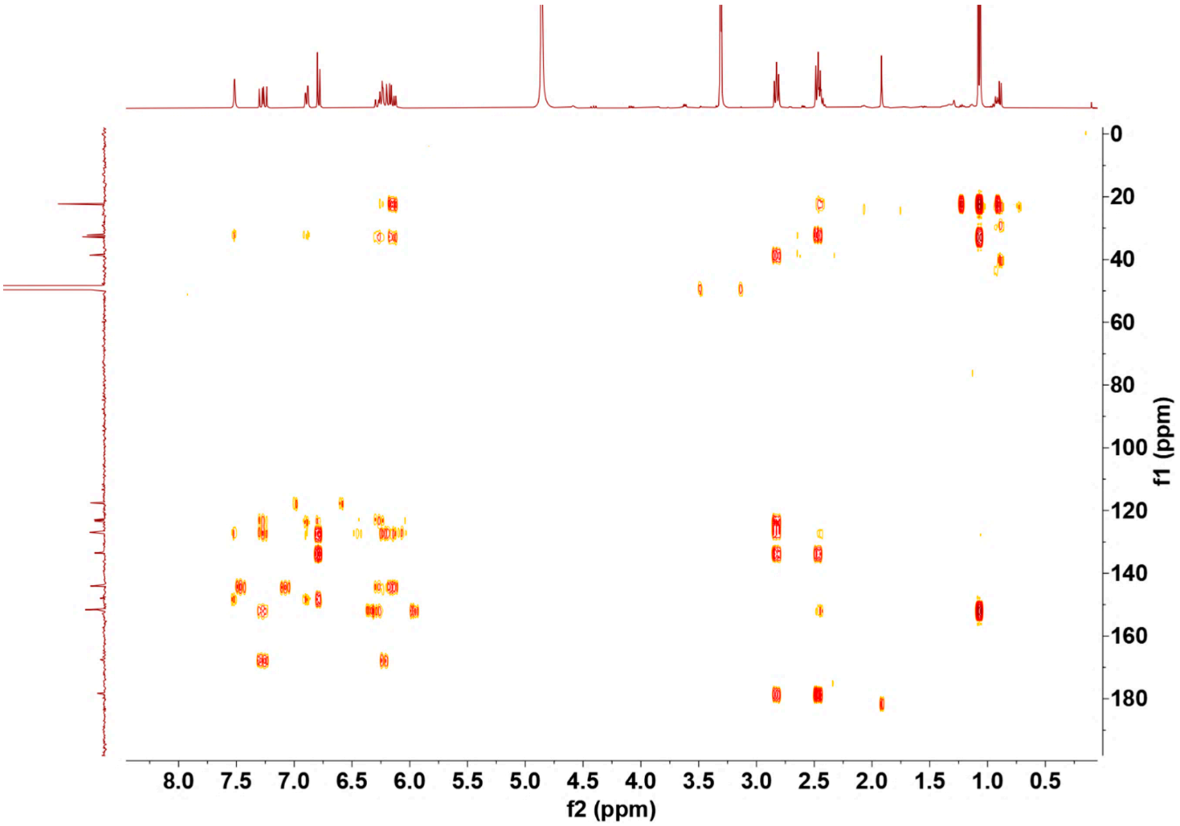

Figure S26. The HMBC spectrum of carpatamide K (**3**) in CD3OD.

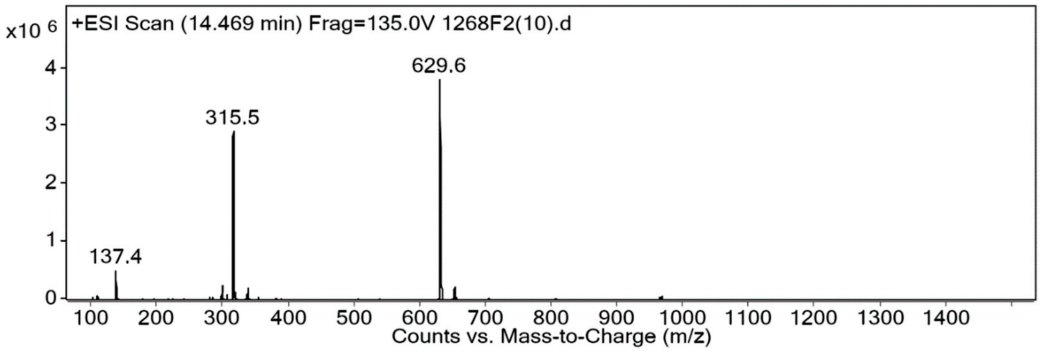

Figure S27. LRESIMS spectrum for carpatamide L (**4**)

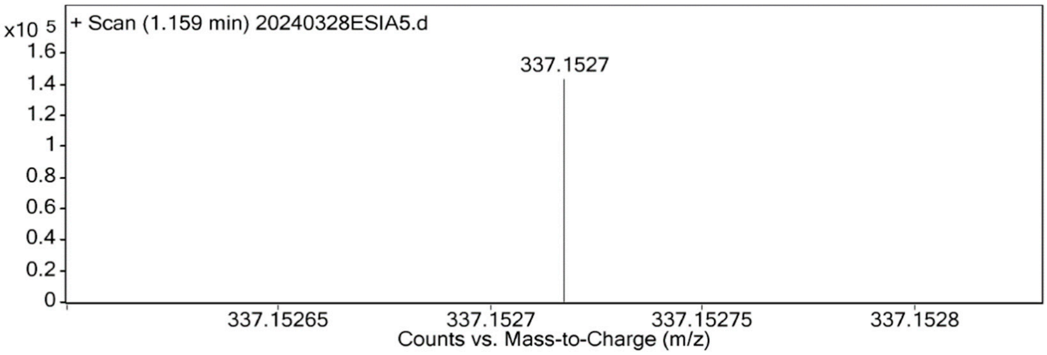

Figure S28. ESI-HRMS spectrum for carpatamide L (**4**)

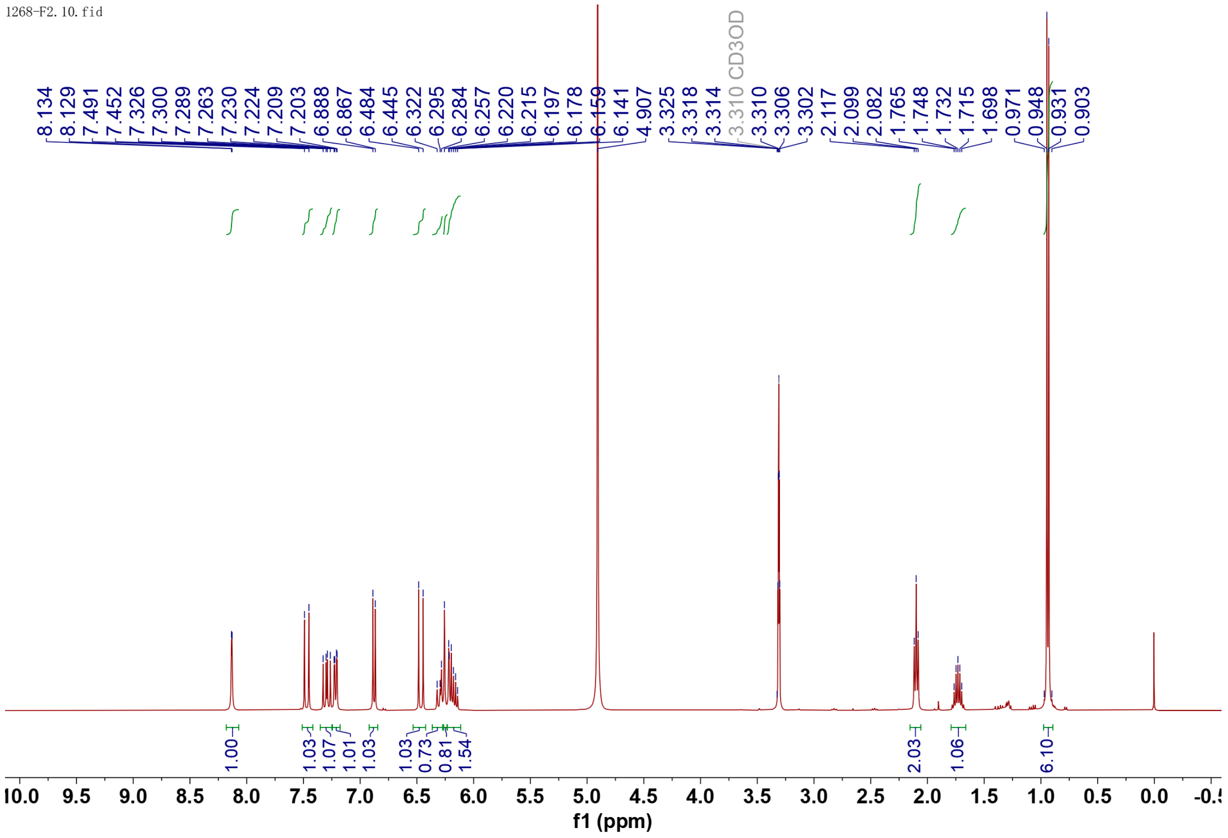

Figure S29. The ¹H NMR (400 MHz) spectrum of carpatamide L (**4**) in CD3OD.

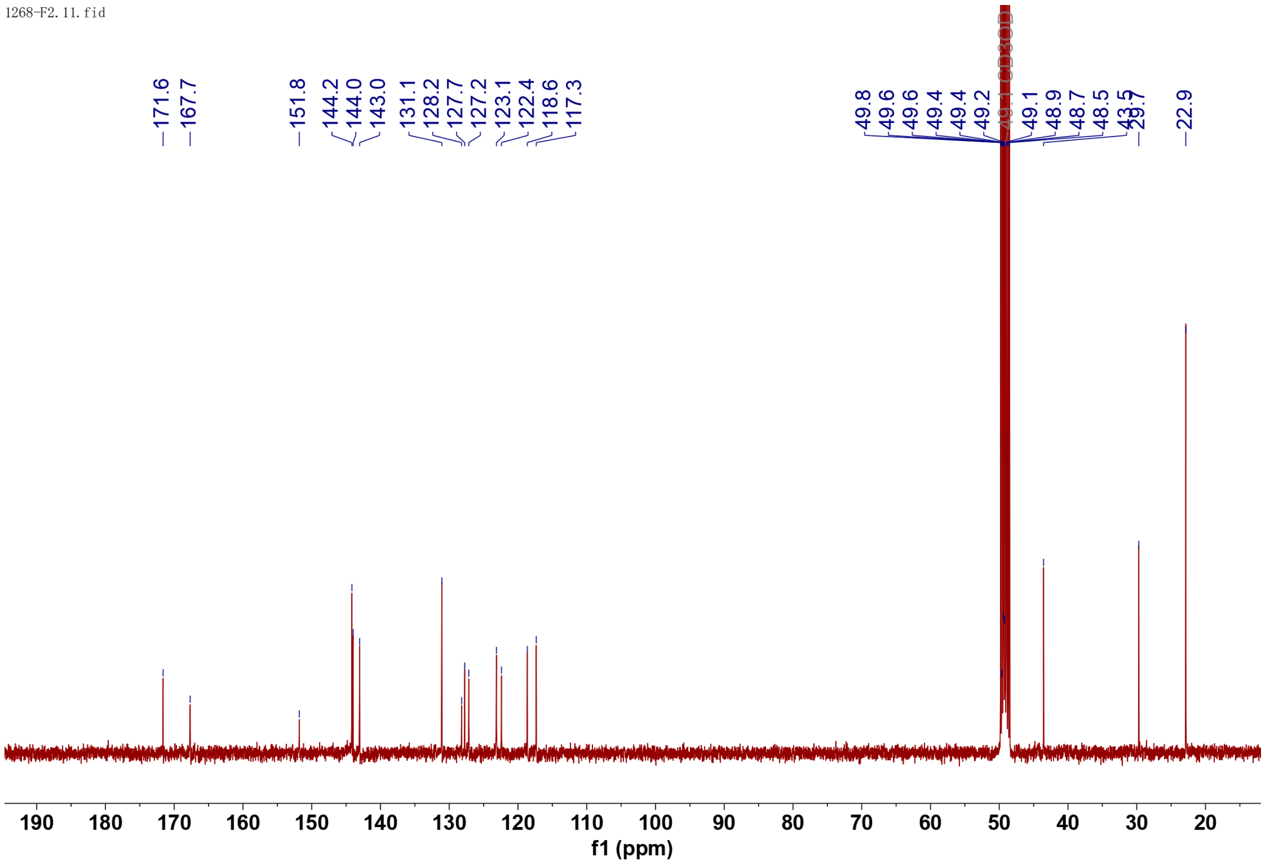

Figure S30. The ¹³C NMR (100 MHz) spectrum of carpatamide L (**4**) in CD3OD.

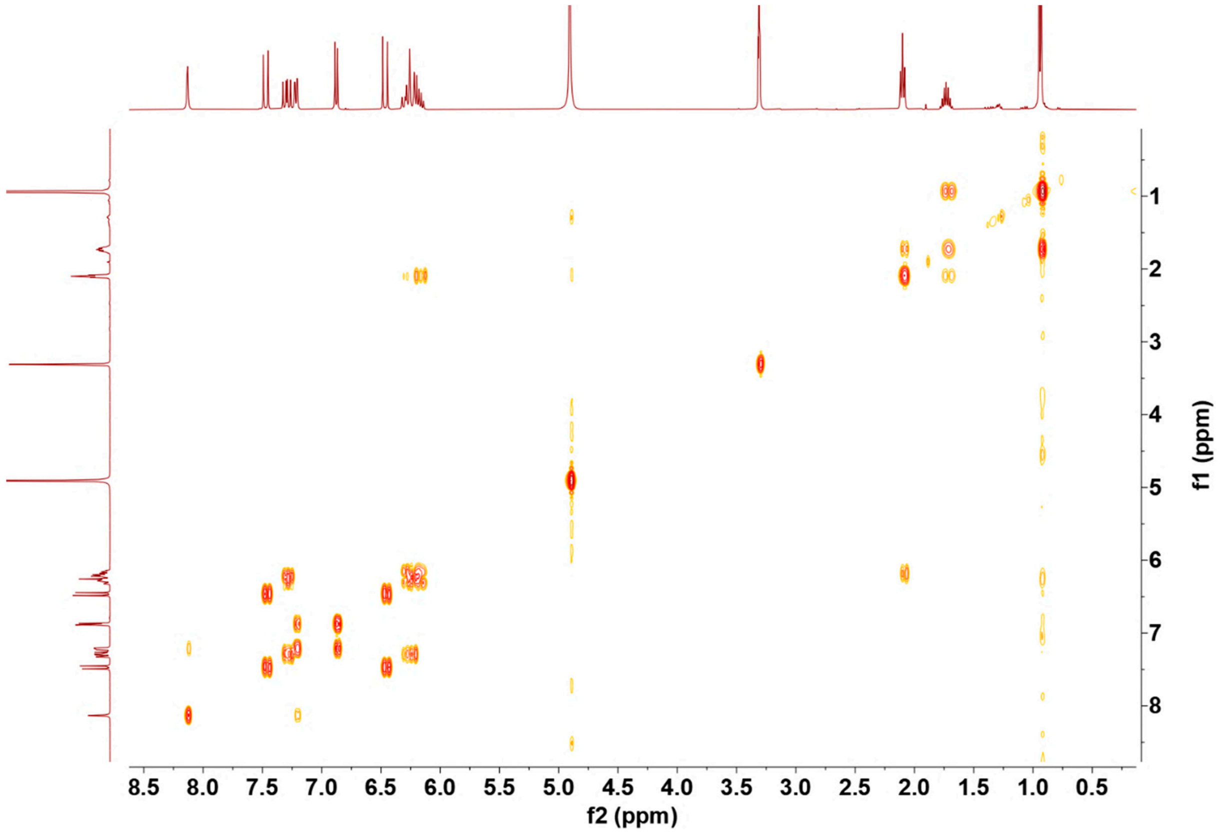

Figure S31. The ^{1}H-^{1}H COSY spectrum of carpatamide L (**4**) in CD_3OD.

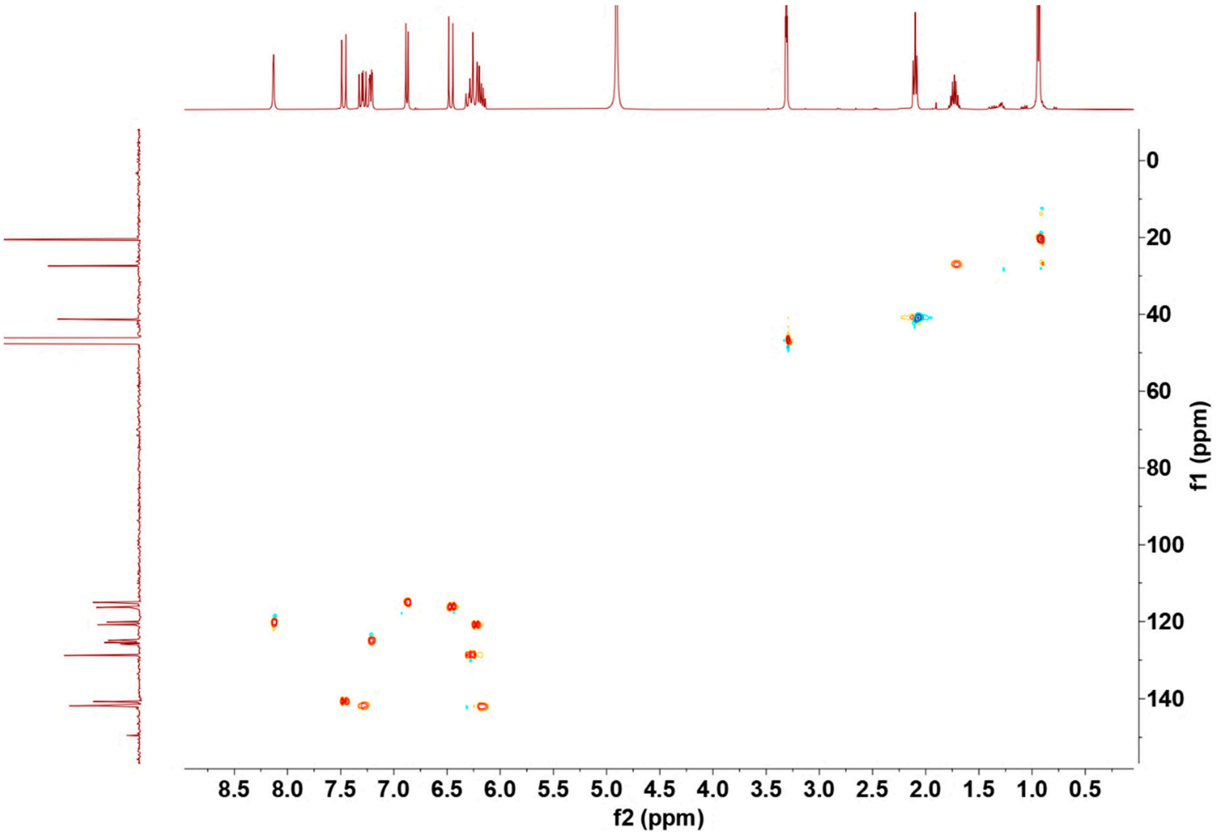

Figure S32. The HSQC spectrum of carpatamide L (**4**) in CD_3OD.

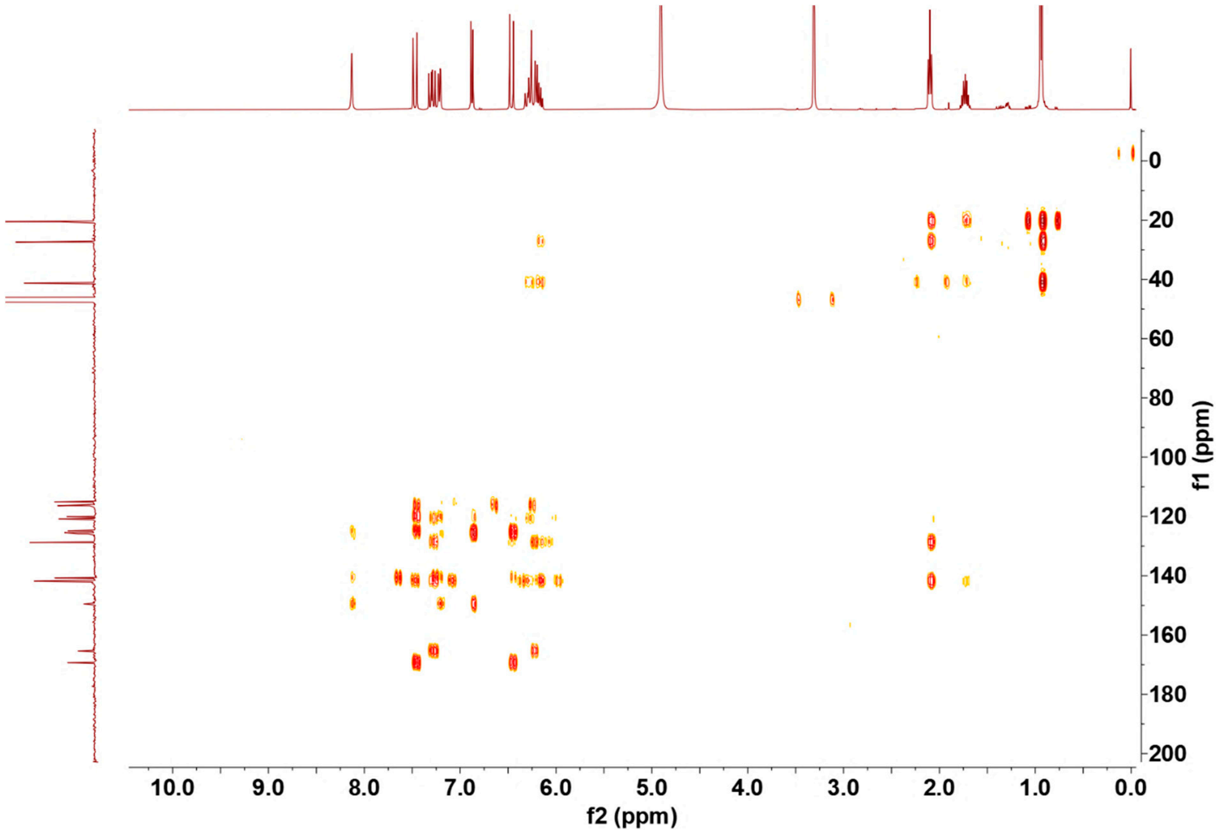

Figure S33. The HMBC spectrum of carpatamide L (**4**) in CD_3OD.

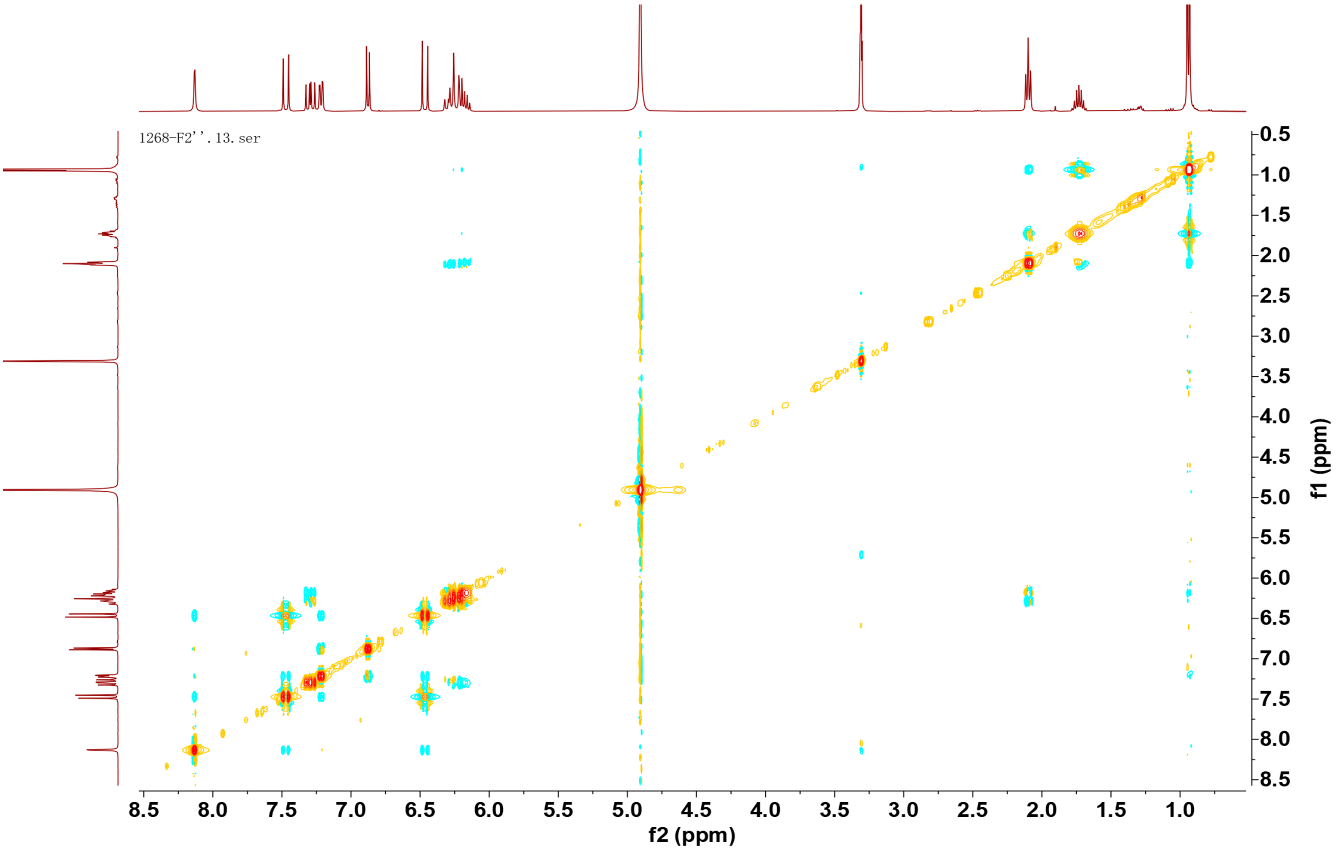

Figure S34. The NOESY spectrum of carpatamide L (**4**) in CD_3OD.

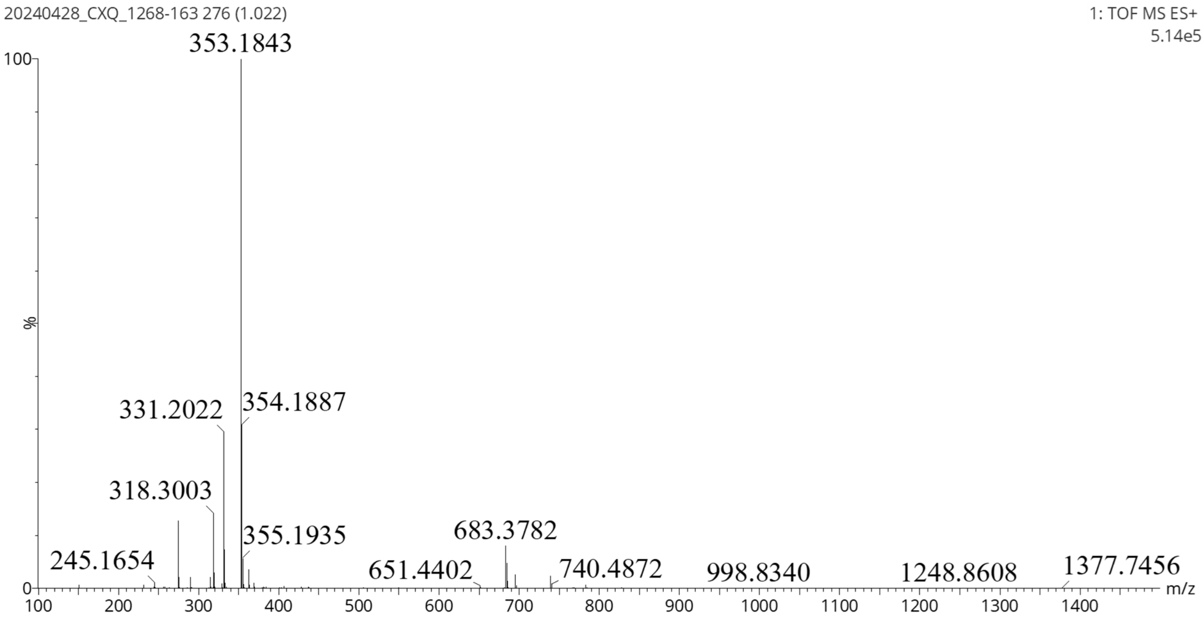

Figure S35. ESI-HRMS spectrum for carpatamide M (**5**)

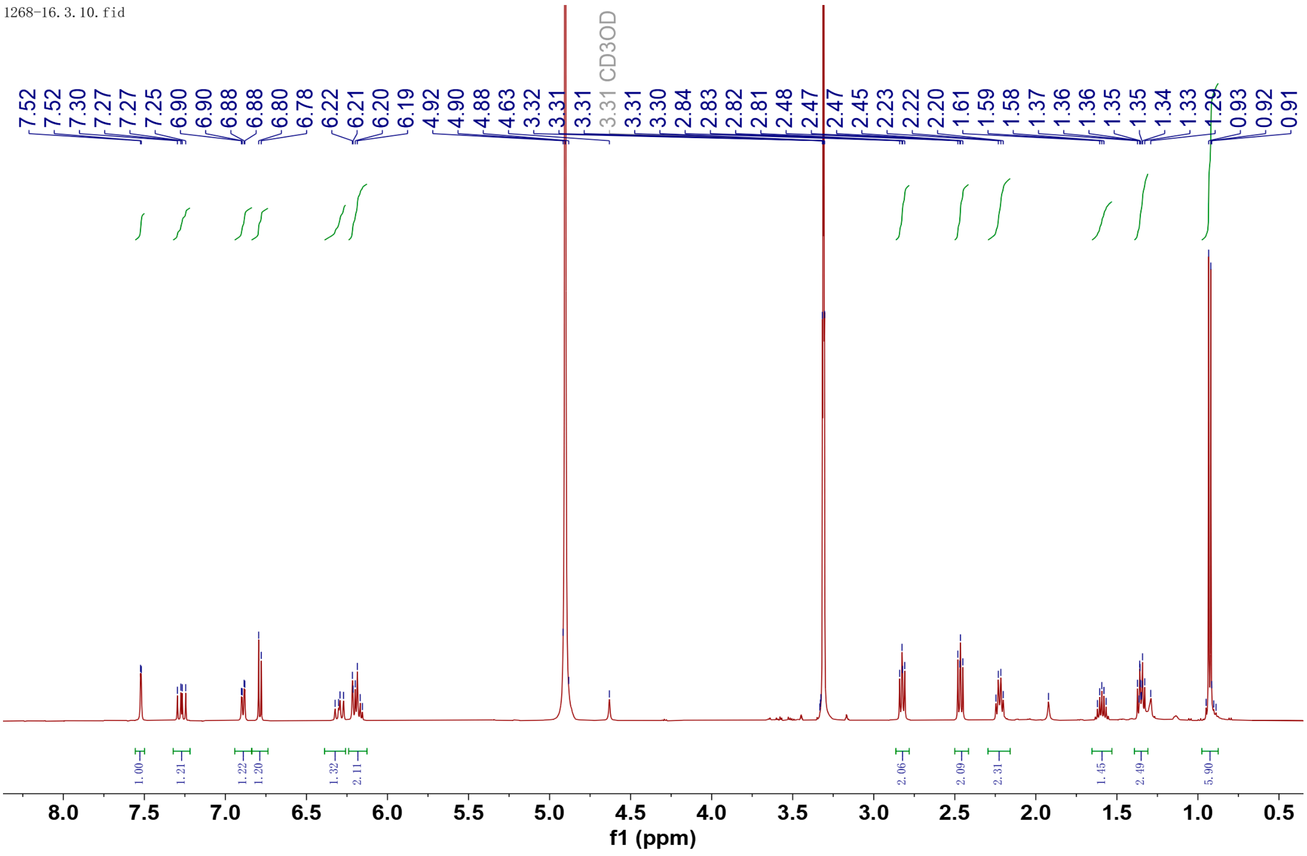

Figure S36. The ^{1}H NMR (400 MHz) spectrum of carpatamide M (**5**) in CD_3OD.

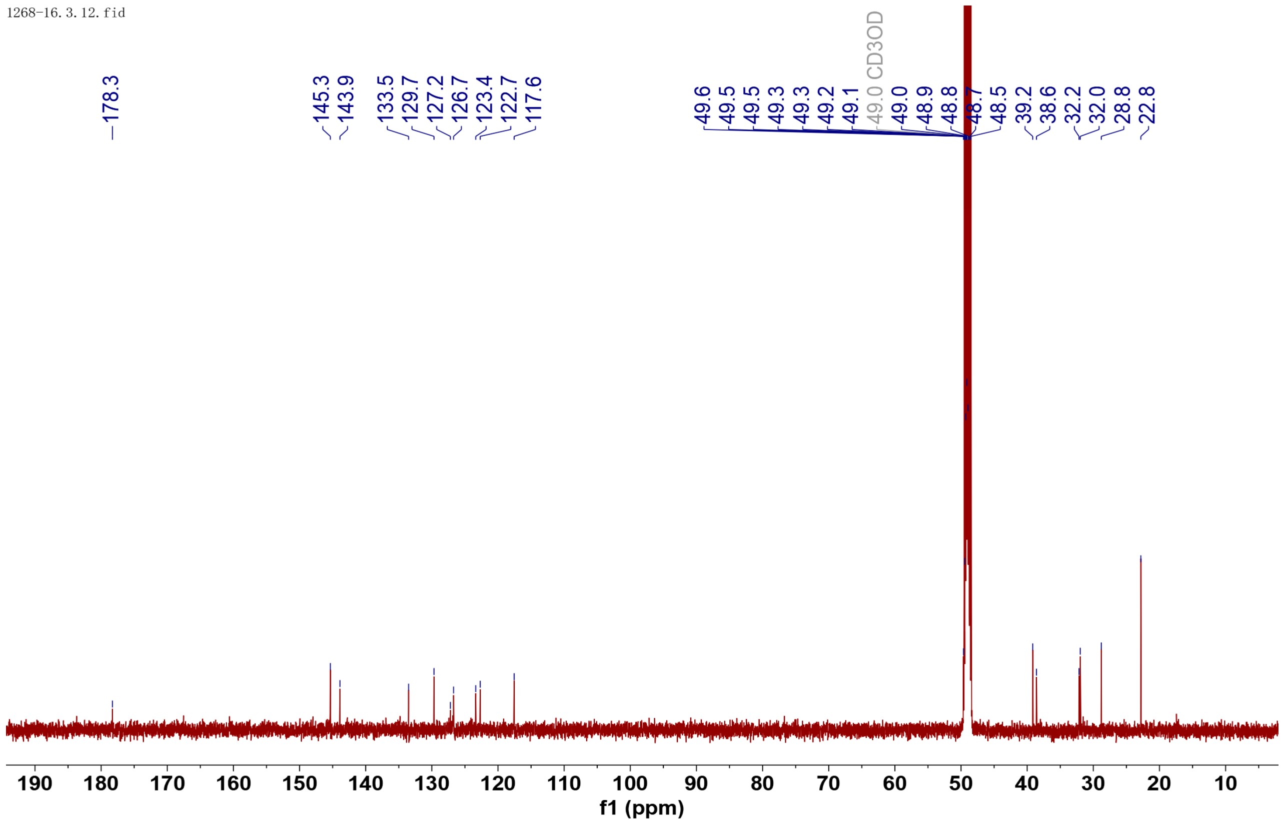

Figure S37. The ^{13}C NMR (100 MHz) spectrum of carpatamide M (**5**) in CD_3OD.

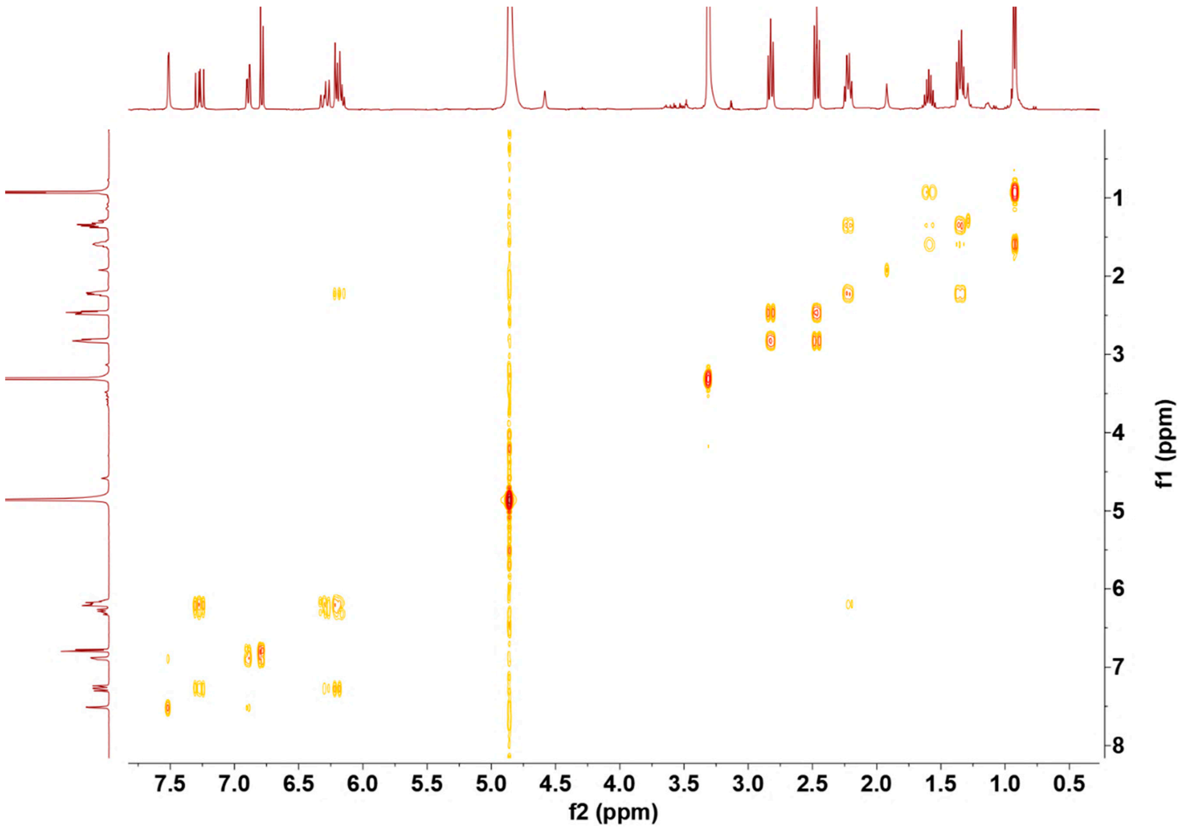

Figure S38. The ^{1}H-^{1}H COSY spectrum of carpatamide M (**5**) in CD_3OD.

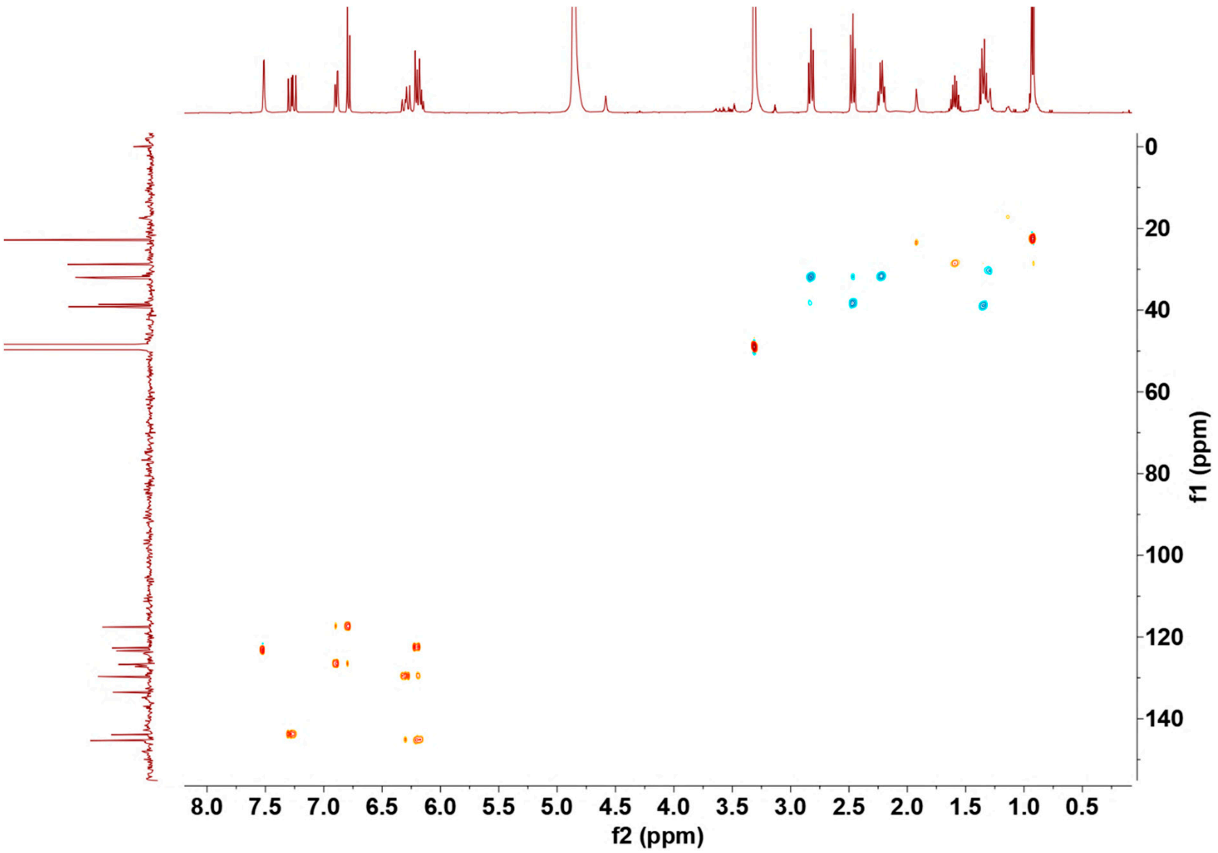

Figure S39. The HSQC spectrum of carpatamide M (**5**) in CD_3OD.

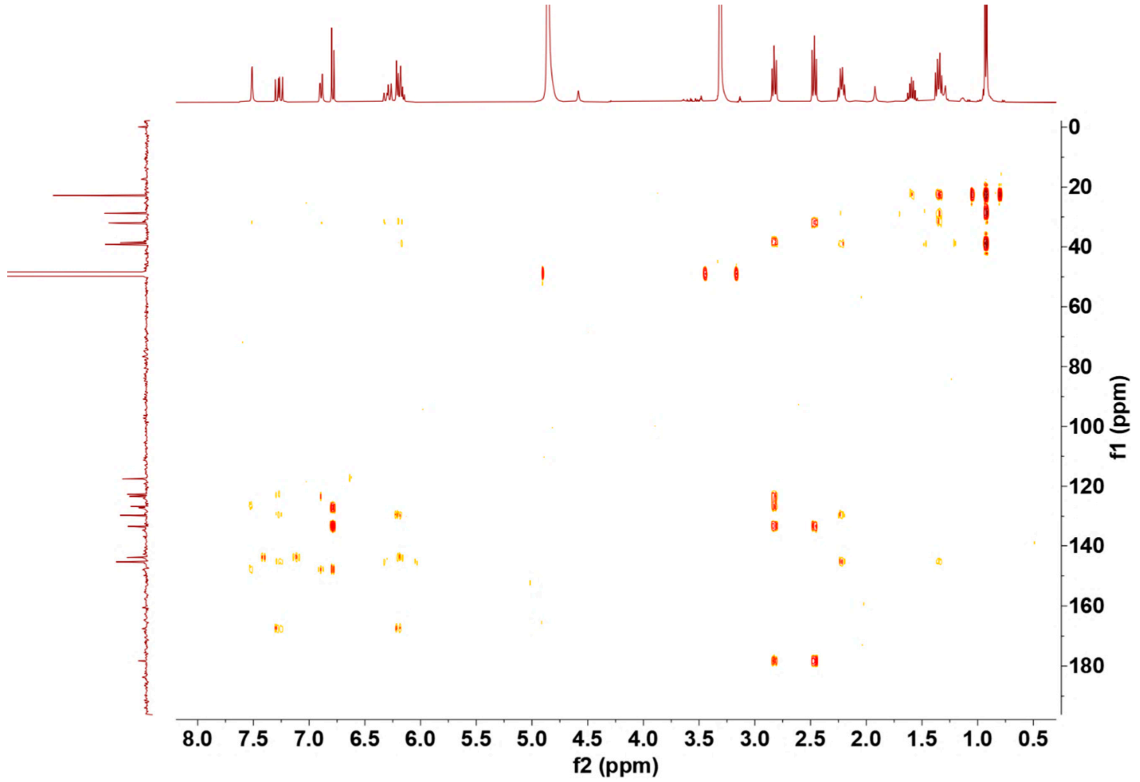

Figure S40. The HMBC spectrum of carpatamide M (**5**) in CD_3OD.

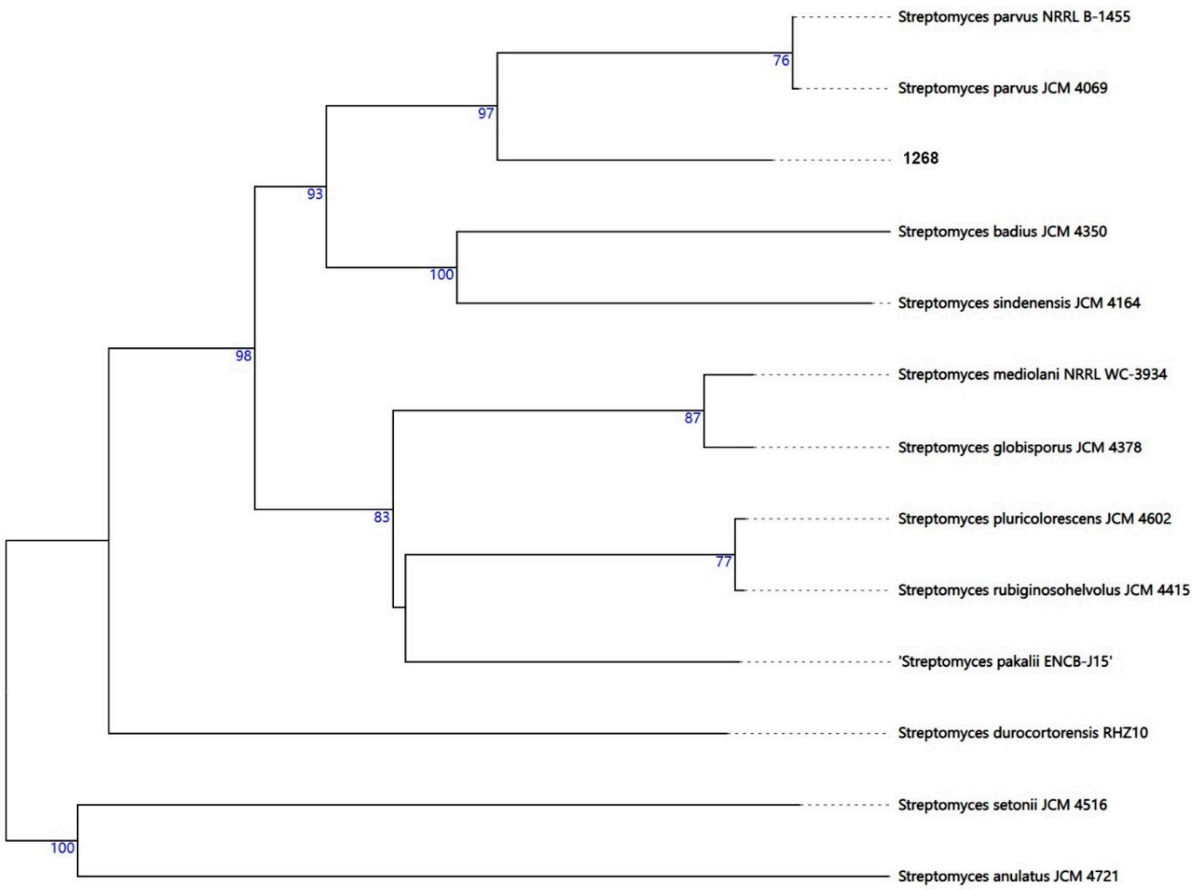

Figure S41. Phylogenetic analysis of the *Streptomyces parvus* 1268.

References

16. Xie, Y.; Li, Q.; Song, Y.; Ma, J.; Ju, J. Involvement of SgvP in carbon-sulfur bond formation during griseoviridin biosynthesis. *ChemBioChem* **2014**, *15*, 1183–1189.
